# The Sputum Microbiome in Pulmonary Tuberculosis and Its Association With Disease Manifestations: A Cross-Sectional Study

**DOI:** 10.3389/fmicb.2021.633396

**Published:** 2021-08-20

**Authors:** Monica R. Ticlla, Jerry Hella, Hellen Hiza, Mohamed Sasamalo, Francis Mhimbira, Liliana K. Rutaihwa, Sara Droz, Sarah Schaller, Klaus Reither, Markus Hilty, Inaki Comas, Christian Beisel, Christoph D. Schmid, Lukas Fenner, Sebastien Gagneux

**Affiliations:** ^1^Swiss Tropical and Public Health Institute, Basel, Switzerland; ^2^University of Basel, Basel, Switzerland; ^3^Swiss Institute of Bioinformatics, Lausanne, Switzerland; ^4^Ifakara Health Institute, Dar es Salaam, Tanzania; ^5^Institute for Infectious Diseases, University of Bern, Bern, Switzerland; ^6^Tuberculosis Genomics Unit, Biomedicine Institute of Valencia, Valencia, Spain; ^7^Department of Biosystems Science and Engineering, ETH Zurich, Basel, Switzerland; ^8^Institute of Social and Preventive Medicine, University of Bern, Bern, Switzerland

**Keywords:** tuberculosis, airway microbiome, sputum, clinical phenotype, chest X-ray, BMI, anaerobes, HIV-TB coinfection

## Abstract

Each day, approximately 27,000 people become ill with tuberculosis (TB), and 4,000 die from this disease. Pulmonary TB is the main clinical form of TB, and affects the lungs with a considerably heterogeneous manifestation among patients. Immunomodulation by an interplay of host-, environment-, and pathogen-associated factors partially explains such heterogeneity. Microbial communities residing in the host's airways have immunomodulatory effects, but it is unclear if the inter-individual variability of these microbial communities is associated with the heterogeneity of pulmonary TB. Here, we investigated this possibility by characterizing the microbial composition in the sputum of 334 TB patients from Tanzania, and by assessing its association with three aspects of disease manifestations: sputum mycobacterial load, severe clinical findings, and chest x-ray (CXR) findings. Compositional data analysis of taxonomic profiles based on 16S-rRNA gene amplicon sequencing and on whole metagenome shotgun sequencing, and graph-based inference of microbial associations revealed that the airway microbiome of TB patients was shaped by inverse relationships between *Streptococcus* and two anaerobes: *Selenomonas* and *Fusobacterium*. Specifically, the strength of these microbial associations was negatively correlated with Faith's phylogenetic diversity (PD) and with the accumulation of transient genera. Furthermore, low body mass index (BMI) determined the association between abnormal CXRs and community diversity and composition. These associations were mediated by increased abundance of *Selenomonas* and *Fusobacterium*, relative to the abundance of *Streptococcus*, in underweight patients with lung parenchymal infiltrates and in comparison to those with normal chest x-rays. And last, the detection of herpesviruses and anelloviruses in sputum microbial assemblage was linked to co-infection with HIV. Given the anaerobic metabolism of *Selenomonas* and *Fusobacterium*, and the hypoxic environment of lung infiltrates, our results suggest that in underweight TB patients, lung tissue remodeling toward anaerobic conditions favors the growth of *Selenomonas* and *Fusobacterium* at the expense of *Streptococcus*. These new insights into the interplay among particular members of the airway microbiome, BMI, and lung parenchymal lesions in TB patients, add a new dimension to the long-known association between low BMI and pulmonary TB. Our results also drive attention to the airways virome in the context of HIV-TB coinfection.

## 1. Introduction

Pulmonary TB is the main clinical form of TB; an airborne infectious disease caused by members of the *Mycobacterium tuberculosis* (*MTB*) complex, and the leading cause of death from a single infection. Worldwide during 2018, TB claimed the lives of 1.5 million people and caused 10 million new cases (WHO, 2019) who further spread the disease via coughing or sneezing *MTB*-carrying droplets. These droplets originate in lung lesions that formed after inhaled *MTB* bacilli reached the alveoli at the end of the lower airways, subverted local immunity, replicated inside infected alveolar macrophages, and triggered inflammatory responses with concomitant lung damage. Lung lesions might be asymptomatic (latent TB) or might progress to more extended lung tissue damage with formation of consolidations and/or cavities and accompanied of signs and symptoms (active TB). Signs and symptoms for TB include weight loss, fever, night sweats, and productive coughing which is required for sustained TB transmission. Latent and active TB are opposite ends in a more complex spectrum of infection outcomes and disease manifestations (Lin and Flynn, 2018).

During active pulmonary TB, patients display considerable inter-individual variability on multiple aspects of disease manifestations: including symptoms, the extent of lung damage, and in the characteristics of lung lesions (Lenaerts et al., 2015); together they are complementary indicators of disease severity. Our understanding of what determines such a wide heterogeneity in TB disease manifestations/severity is still incomplete. However, evidence suggests an interplay of factors associated with the host, the pathogen, and the environment (Gagneux et al., 2006; Chandrasekaran et al., 2017; Bastos et al., 2018). Among these factors, the microbial communities inhabiting the host's respiratory tract (i.e., the airways microbiome) have the potential to improve our yet limited understanding of pulmonary TB (Naidoo et al., 2019).

The respiratory tract environment not only experiences shifts in physico-chemical and immunological conditions during diseased states but also shifts in the composition of resident microbial communities. These microbial communities are not merely a reflection of local physiological conditions (Quinn et al., 2018a, 2019), they modulate inflammatory responses which mediate lung injury, and are associated with disease severity and mortality (Wu and Segal, 2017). For instance, patients with severe asthma have bronchial airways enriched with *Actinobacteria* and *Klebsiella* species (Huang et al., 2015), and 1-year mortality of hospitalized COPD patients was associated with baseline sputum microbial composition (Leitao Filho et al., 2019).

In pulmonary TB, evidence of the relationship between resident microbial communities of the distinct compartments of the respiratory tract (i.e., airway microbiome) and disease severity is limited to three studies; one in rhesus macaques (Cadena et al., 2018) and the other two in humans (Zhou et al., 2015; Nakhaee et al., 2018). The comparability of their results is not only limited by the different host types but also by different types of respiratory samples and different definitions of disease severity. Definitions of disease severity included the degree of pulmonary inflammation, lung side involvement (Cadena et al., 2018), presence/absence of lung lesions (Zhou et al., 2015), and clinical symptoms (Nakhaee et al., 2018).

Given the scant knowledge of the airway microbiome in TB patients and of the relationship with disease severity, we performed a large cross-sectional study of patients with active pulmonary TB from a high TB burden setting, Dar es Salaam (Tanzania). For this human cohort, we characterized the microbial composition of expectorated sputum by 16S-rRNA-gene amplicon (16S-A) and whole-metagenome shotgun (WMS) sequencing, and aimed at identifying biomarkers associated with TB severity. Instead of using a single definition of disease severity, we preferred to investigate associations with multiple aspects of disease manifestations, which encompass complementary forms of disease severity. Thus, we investigated associations with mycobacterial load in the sputum, radiographic signs (chest x-rays), and clinical findings (signs and symptoms). We controlled for the potential effects of sex, age, physical health status (underweight, anemia, smoking, and alcohol abuse), season, and co-infections with HIV, respiratory pathogens, and helminths.

This study presents a comprehensive investigation of the hypothesis that inter-individual variability of the airway microbiome in TB patients is associated with differences in TB-disease manifestations; specifically aiming at identifying biomarkers associated with TB-disease severity in a non-invasively collected respiratory sample, the expectorated sputum.

## 2. Materials and Methods

### 2.1. Study Setting

We included TB patients from an ongoing prospective cohort that studies the clinical and molecular epidemiology of TB in the Temeke district of Dar es Salaam, Tanzania (TB-DAR). Tanzania is among the top 20 countries with the highest TB incidence; 142,000 new TB cases were estimated in Tanzania during 2018, 28% of them were co-infected with HIV (WHO, 2019). The city of Dar es Salaam has the highest TB incidence in the country (20 % of TB cases notified during 2018) (NTLP, 2018). The Temeke district is a densely populated urban setting accounting for one third of the TB cases from Dar es Salaam (Said et al., 2017).

### 2.2. Study Population and Procedures

We conducted a cross-sectional study nested within the ongoing TB-DAR cohort. The study population and the procedures of this cohort have been previously described in detail (Mhimbira et al., 2016, 2017, 2019; Steiner et al., 2016; Hiza et al., 2017; Said et al., 2017; Hella et al., 2018; Sikalengo et al., 2018). Briefly, at the Temeke district hospital and since November 2013, TB-DAR have been recruiting sputum smear positive or Xpert MTB/RIF positive adult TB patients (≥18 years of age). TB was further confirmed by sputum culture in Lwenstein-Jensen (LJ) solid media; clinical isolates were further analyzed by lineage-specific allele probes in real-time PCR for singleplex SNP-typing according to standard protocols (Applied Biosystems, Carlsbad, USA) and as previously described (Stucki et al., 2018). In this study, we only included newly diagnosed TB patients, without previous history or diagnosis of TB, recruited between November 2013 and November 2015. Included TB cases were randomly chosen to obtain a representative subset of the entire cohort during the recruitment period.

At the time of TB diagnosis, participants were interviewed to collect data on socio-demographic characteristics, lifestyle, symptoms, previous use of medications, and health-seeking behavior (Said et al., 2017). Patients underwent physical and chest X-ray examination (CXR). Before initiation of TB treatment, biological specimens (sputum, naso-pharyngeal swabs, blood, urine, and stool) were collected for further investigation of anemia and co-infections, as previously described (Mhimbira et al., 2017, 2019; Hella et al., 2018). Investigated co-infections included HIV (Mhimbira et al., 2017; Hella et al., 2018), helminths (Mhimbira et al., 2017), and respiratory pathogens (Mhimbira et al., 2019). For HIV-positive patients, CD4+ T cell counts were also obtained (Mhimbira et al., 2017).

### 2.3. Collection of Sputum Samples and DNA Extraction

To ensure comparability and quality of sputum samples, health-care workers provided video-guided instructions to all participants and asked them to only submit specimens collected during early-morning. The use of the sputum submission instructional video, for improvement of sputum quality, was previously validated by Mhalu and colleagues (Mhalu et al., 2015). At the time of specimen reception, laboratory technicians visually assessed quality and volume; second samples were requested if salivary-like (transparent and watery specimen with bubbles) specimens were submitted. Accepted sputum samples were transported from Temeke district hospital at 4°C to the Bagamoyo Research and Training Center for processing. Total DNA was extracted from sputum specimens using the QIAamp DNA Kit (Qiagen, Germany) according to the supplier's instructions (Spin Protocol for DNA purification from Blood or Body Fluids).

### 2.4. Amplicon Sequencing

We followed Illumina's 16S amplicon sequencing library preparation protocol (Illumina, 2013). The protocol included: (i) PCR amplification of the V3-V4 region of the 16S rRNA gene with primers PCR1_Forward (50 bp, 5′-TCGTCGGCAGCGTCAGATGTGTATAAGAGACAGCCTACGGGNGGCWGCAG-3′), and PCR1_Reverse (55 bp, 5′-GTCTCGTGGGCTCGGAGATGTGTATAAGAGACAGGACTACHVGGGTATCTAATCC-3′); (ii) library preparation with the Nextera XT Index Kit, and (iii) paired-end sequencing (2 × 300 bp, v3 chemistry) on the MiSeq platform (Illumina, San Diego, CA), samples were distributed across three sequencing runs. To account for potential sequencing batch effects, we included the sequencing run number as a co-factor in multivariate models.

### 2.5. Whole Metagenome Shotgun Sequencing

DNA samples were first selected based on their quality (limited degradation and high concentration) as assessed by DNA gel electrophoresis (1:10 DNA dilutions run on 1% agarose gels at 90 volts for 45 min). DNA concentration was determined by PicoGreen quantification with Qubit and normalized to 0.2 ng/ul. Dual-indexed paired-end libraries were prepared using the Nextera XT DNA Library Prep kit; the standard protocol was followed at the sequencing facility of FISABIO (Foundation for the Promotion of Health and Biomedical Research in the Valencian Community). Paired-end sequencing (2 × 150 bp) on the HiSeq 2,500 platform was done at the Department of Biosystems Science and Engineering (D-BSSE) of ETH Zeich. To minimize batch effects, we used three sequencing runs to repeatedly sequence all samples and sample pools per lane combined samples picked in a random order. Background controls (water) and a mock community (20 strain even mix genomic material, ATCC® MSA-1002™) were included during the entire workflow.

### 2.6. Data Definitions

Newly diagnosed TB cases were defined as TB patients that never received TB treatment or received TB treatment for <1 month. We further stratified those patients by multiple aspects of disease manifestations: mycobacterial load in their sputum, severity of clinical findings (signs and symptoms), and presence of radiologic signs (lung parenchymal infiltrates, cavities, lymphadenopathy, micronodules, pleural efussion, etc.) in chest X-rays (CXRs).

To define sputum mycobacterial load as low or high, we used sputum acid-fast bacilli (AFB) smear results. Following the World Health Organization/International Union Against Tuberculosis and Lung Disease (WHO/IUATLD) guidelines, sputum AFB smear results were expressed as quantitative categories (“Scanty,” “1+,” “2+,” and “3+”) that grade the number of AFB per number of microscopic fields (Mhimbira et al., 2017). As previously described (Mhimbira et al., 2019), we defined sputum mycobacterial burden as high if AFB smear results were “2+” (1–10 AFB per field, 50 fields) or “3+” (> 10 AFB per field, 20 fields); and as low if AFB smear results were “Scanty” (1–9 AFB in 100 fields) or “1+” (10–99 AFB in 100 fields).

**Figure 1:**
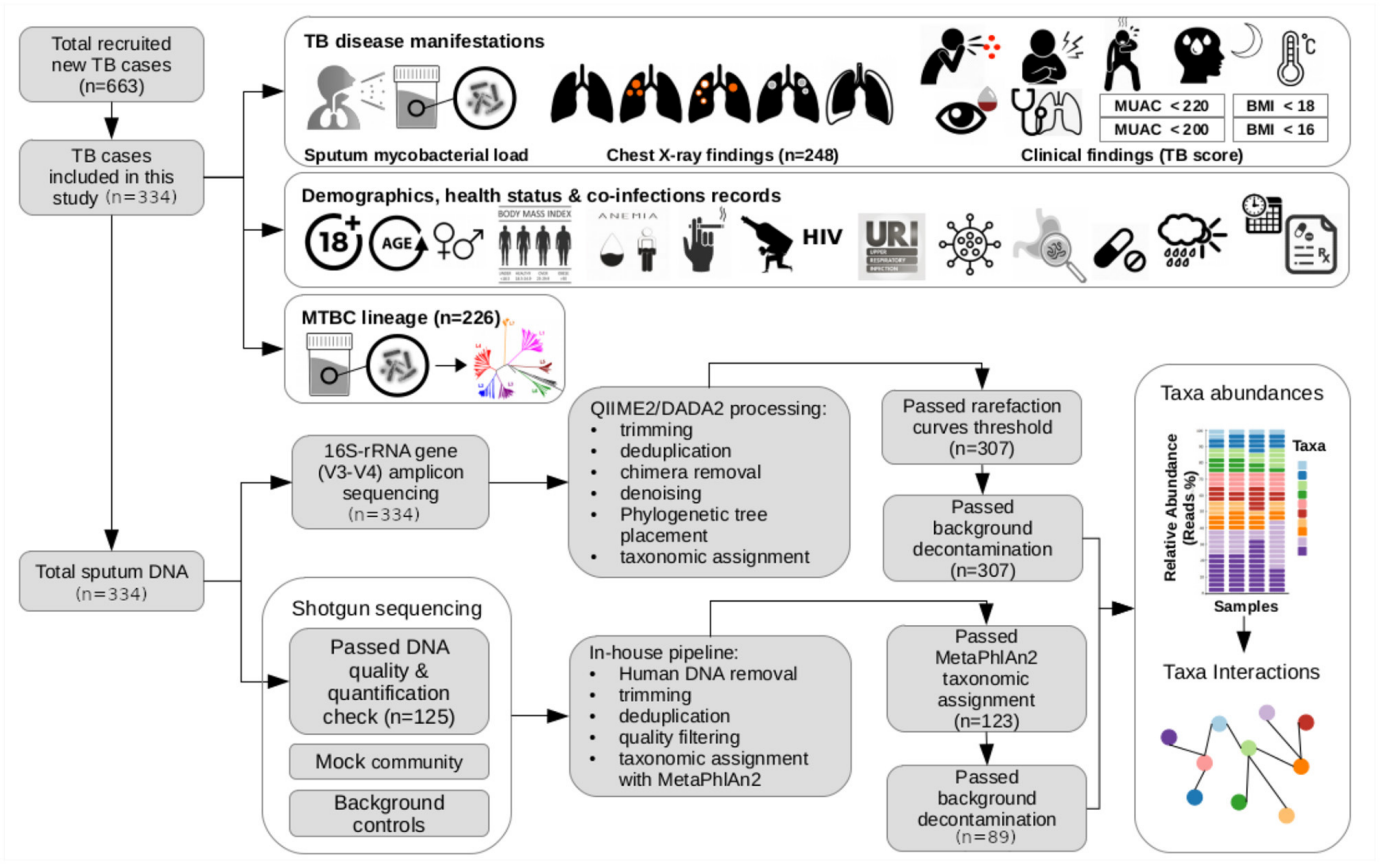
Schematic summary of the study. In this study, we included 334 participants of the newly diagnosed adult TB patients (≥ 18 years old) recruited within November 2013 and November 2015 for an ongoing prospective cohort study (TB-DAR). TB-DAR studies the clinical and molecular epidemiology of TB in the Temeke district of Dar es Salaam, Tanzania. Baseline clinical and demographic characteristics were retrieved for each TB patient. Early-morning expectorated sputum samples were collected to characterize the taxonomic microbial composition by targeted 16S-rRNA-gene amplicon sequencing (16S-AS) and whole-metagenome shotgun sequencing (WMS-S). Briefly, the data analysis workflow is shown. We assessed the potential association of the sputum microbial composition with three aspects of TB disease manifestations: (i) mycobacterial load in the sputum; (ii) chest X-rays findings; and (iii) severity of clinical findings, estimated by a TB disease score considering signs and symptoms for TB. As shown in the figure, top right, clinical findings included cough, haemoptysis (i.e., expectoration of blood), chest pain, dyspnoea (i.e., shortness of breath), night sweating, fever, anemic conjuctivae, positive finding at lung auscultation, MUAC <220 mm, MUAC <200 mm, BMI <18 kg m-2, and BMI <16 kg m-2. BMI, Body Mass Index; MUAC, Mid-Upper Arm Circumference: URI, Upper Respiratory Infection.

To define clinical findings for pulmonary tuberculosis as mild or severe, we adapted the TB score (Mhimbira et al., 2017) defined by Wejse and colleagues (Wejse et al., 2008). The TB score was expressed as cumulative points (from 0 to 12) that quantify the number of clinical findings observed in a patient during physical examination. Twelve clinical findings were considered, each one counted as one if present: (i) cough, (ii) haemoptysis, (iii) dyspnoea, (iv) chest pain, (v) night sweating, (vi) anemic conjuctivae, (vii) positive finding at lung auscultation (crepitation, rhonci, subdued, or complete absence of respiratory sounds), (viii) fever (temperature > 37°C), (ix) mid upper arm circumference (MUAC) <220 mm, (x) MUAC <200 mm, (xi) body mass index (BMI) <18 kg m-2, and (xii) BMI <16 kg m-2 (see Figure 1). As in Mhimbira and colleagues (Mhimbira et al., 2017), we defined clinical findings for pulmonary tuberculosis as severe if the TB score was ≥6; and as mild, otherwise. To screen for lung abnormalities, double readings of CXRs were performed by board-certified radiologists, and discrepancies were resolved by an independent reader.

To account for differences in disease manifestations due to delay in diagnosis, duration of diagnostic delay was included based on the longest reported TB-related symptom and categorized into: ≤ 3 weeks and >3 weeks, as previously described by Said and colleagues (Said et al., 2017). Differences in clinical manifestations might be associated to differences in the genetic background of *MTB*, thus we included the SNP-typing results of the *MTB* clinical isolates.

Socio-demographic variables included sex and age. Physical health status parameters included BMI, hemoglobin (Hb) levels (kg dL-1), smoking, and alcohol abuse. To assess adult nutritional status, we followed WHO's BMI-based definitions (Bailey and Ferro-Luzzi, 1995), as follows: (i) “Underweight” (BMI <18.5), (ii) “Normal weight” (BMI, 18.5–24.9), and iii) “Obesity” (BMI ≥ 25). Similarly, we followed WHO's Hb cut-offs to define “Non-Anemia” (Hb ≥ 12 for women, and Hb ≥ 13 for men), “Mild” (11–11.9 for women, and 11–12.9 for men), “Moderate” (8.0–10.9 for women and men), and “Severe” (<8.0 for women and men) anemia (WHO, 2011).

### 2.7. Taxonomic Profiling

16S-rRNA-gene amplicon sequencing (hereafter 16S-AS) reads were processed with QIIME2 plugins v2019.7 (Bolyen et al., 2019). We first carried out a quality inspection of paired-end reads with QIIME2's “demux” plugin which revealed reverse reads had poor quality at the 3'end and had to be trimmed off before merging read pairs with DADA2 v1.10 (Callahan et al., 2016); this resulted in a 90% loss of the data (analysis is available at https://git.scicore.unibas.ch/TBRU/tbdarbiome_cases/-/blob/master/notebooks/00_MOT_qiime2-qc-denoising-features_pairedreads.ipynb). Therefore, we decided to only use the forward reads of every pair, which were processed as follows. First, we run the denoise-single method of QIIME2's dada2 plugin, followed default parameter settings but adjusted the number of bases trimmed at the 5′- and 3′- ends. To create a phylogenetic tree of the resulting amplicon sequence variants (ASVs), we run the q2-fragment-insertion plugin which uses the SAT-enabled phylogenetic placement (SEPP, Mirarab et al., 2012) algorithm to insert our ASVs into the reference phylogeny of 16S rRNA gene sequences (Greengenes v13.8, reference sequences clustered at 99% sequence similarity) (Janssen et al., 2018); unplaced ASVs were removed. Finally, we used the *q2-feature-classifier* plugin (Bokulich et al., 2018) to assign taxonomic classifications to our ASVs; we first retrained the classifier with 16S V3-V4 fragments extracted from the Greengenes v13.8 reference sequences. The workflow is available as a Jupyter notebook at https://git.scicore.unibas.ch/TBRU/tbdarbiome_cases/-/blob/master/notebooks/01_MOT_qiime2-qc-dada2_denoising_features_read1.ipynb.

Whole-metagenome shotgun (WMS) reads were processed with an in-house analysis pipeline that performs quality pre-processing, taxonomic profiling, and functional profiling. Quality pre-processing included adapter/quality trimming, and filtering with *fastp* (Chen et al., 2018); removal of reads derived from human DNA with BBTools' (Bushnell, 2014) *bbsplit*; and removal of duplicated reads with BBTools' *clumpify*. Taxonomic profiling was performed with MetaPhlAn2 (Truong et al., 2015). The pipeline is available at https://git.scicore.unibas.ch/TBRU/MetagenomicSnake and details of its execution for this publication are available in a Jupyter notebook at https://git.scicore.unibas.ch/TBRU/tbdarbiome_cases/-/blob/master/notebooks/02_MOT_metasnk-wms-data-processing.ipynb. For each species detected, we obtained the reported mode of metabolism (i.e., oxygen requirement) by manually querying https://bacdive.dsmz.de, https://www.lgcstandards-atcc.org/, https://microbewiki.kenyon.edu/, http://www.homd.org, and Pubmed Central® (PMC); the resulting table is available at https://git.scicore.unibas.ch/TBRU/tbdarbiome_cases/-/blob/master/data/raw/metadata/species_oxygen_tolerance.csv.

### 2.8. Identification of Potential Contaminants

We used R package *decontam* v1.6 (Davis et al., 2018) to identify contaminant DNA sequences. For the 16S-AS dataset, *decontam* used the DNA concentration of sputum samples to identify contaminant ASVs whose frequency varied inversely with total DNA concentration (frequency-based identification). For the WMS dataset, frequency-based identification was complemented with presence of contaminants in background controls (prevalence-based identification) and in a mock community (20 strain even mix genomic material, ATCC® MSA-1002™). The analysis is available at https://git.scicore.unibas.ch/TBRU/tbdarbiome_cases/-/blob/master/notebooks/03_MOT_decontam.ipynb.

### 2.9. Diversity Estimations

To quantify diversity within each sputum sample (alpha diversity), we used the 16S-AS taxonomic profiles to compute Faith's phylogenetic diversity (PD) metric (Faith, 1992), as implemented in QIIME2 v2019.7 (Bolyen et al., 2019). We favored the PD metric as it considers phylogenetic differences between taxa which makes PD to account the limited contribution of closely related taxa to diversity (Matsen, 2015). To minimize the effect of differences in sequencing depth, we computed rarefaction curves to set a threshold for even sampling (i.e., equal number of sequences) across all samples before computing the PD metric. Analysis available at https://git.scicore.unibas.ch/TBRU/tbdarbiome_cases/-/blob/master/notebooks/06_MOT_alpha-rarefaction.ipynb.

To quantify differences in community composition among samples (beta diversity) and identify variability patterns, we computed Aitchison distances which acknowledge the compositional nature of the taxonomic profiles (Gloor et al., 2017; Quinn et al., 2018b). Aitchison distances correspond to Euclidean distances among taxonomic profiles (i.e., taxon abundances) that were centered log-ratio (CLR) transformed. The CLR transformation takes compositional vectors (i.e., relative abundances) from a constrained space (i.e., the unit simplex) to an unconstrained space of logratio vectors (logarithm of the ratio between the abundance of a taxon in a sample and the geometric mean of all taxon abundances in the same sample). Resulting CLR-transformed vectors are scale-invariant and subcompositionally coherent which implies that rarefying the taxonomic profiles to a constant value across samples will not have significant effects on the relationships between samples and between taxa; therefore we computed Aitchison distances on non-rarefied taxonomic profiles. To circumvent the limitation of undefined values when computing the logarithm of zero values, zero abundances were replaced following Martin-Fernndez et al.'s Bayesian-multiplicative replacement strategy (Martín-Fernández et al., 2014); implemented in *cmultRepl* of the R package *zCompositions* v1.3.4. To visualize community composition profiles across samples, we created heatmaps with sputum samples and taxa sorted according to agglomerative hierarchical clustering based on Aitchison distances; analysis available at https://git.scicore.unibas.ch/TBRU/tbdarbiome_cases/-/blob/master/notebooks/08_MOT_taxonomic-summaries.ipynb. To visualize relationships among taxa across sputum samples, we created relative variation biplots following Aitchison and colleagues rules for creation and interpretation of such biplots (Aitchison and Greenacre, 2002). Analysis is available at https://git.scicore.unibas.ch/TBRU/tbdarbiome_cases/-/blob/master/notebooks/10_MOT_inference-of-genus-genus-interactions.ipynb.

### 2.10. Compositional Network Reconstruction

To infer associations among taxa, we created genus-level and species-level interaction networks with SParse InveresE Covariance Estimation for Ecological ASociation Inference (SPIEC-EASI); available as an R package (*SpiecEasi v1.0.7*). SPIEC-EASI uses CLR-transformed abundances to infer a graph model where nodes represent taxa and edges represent associations between taxa that cannot be explained by alternative paths in the graph (Kurtz et al., 2015). We selected the neighborhood selection method of SPIEC-EASI. Inferred interactions were also confirmed by Spearman's rank-order correlation. Additionally, we also used the SparCC method (Friedman and Alm, 2012), as implemented in the R package *SpiecEasi*.

To infer species-level interaction networks, we used the WMS-S taxonomic profiles and considered only those species present in at least 3 samples. To infer genus-level interaction networks, we used the 16S-AS taxonomic profiles and considered only those genera present in at least 5% of the samples included in the dataset; genera are more likely to be shared across samples, thus we increased the presence threshold. Analyses are available at https://git.scicore.unibas.ch/TBRU/tbdarbiome_cases/-/blob/master/notebooks/10_MOT_inference-of-genus-genus-interactions.ipynb and at https://git.scicore.unibas.ch/TBRU/tbdarbiome_cases/-/blob/master/notebooks/11_MOT_inference-of-spp-spp-interactions.ipynb.

### 2.11. Statistical Analysis

We performed statistical analysis with the R software environment v3.6. Associations of categorical demographic and clinical characteristics with TB disease manifestations were assessed by chi-squared test or Fisher's exact test if expected frequencies were below five. Associations with continuous variables were assessed by student *t*-tests or Wilcoxon rank-sum test when normality could not be assumed; normality was evaluated with Shapiro-Wilk test and Q-Q plots. Single test significance level was Bonferroni adjusted for multiple comparisons. Analyses are available at https://git.scicore.unibas.ch/TBRU/tbdarbiome_cases/-/blob/master/notebooks/07_MOT_characteristics-of-cohort.ipynb.

To test associations of Faith's PD with TB disease manifestations (mycobacterial load, severity of clinical findings, and peresence of radiologic signs), we used the R-package *car* v3.0 to create two multi-way ANCOVA models with log transformed Faith's PD as response variable. With the first ANCOVA model, we simultaneously assessed the marginal effects of abnormal CXR-findings and high mycobacterial load of sputum; a decision based on previous analysis indicating that these clinical manifestations were not associated with each other. The model was adjusted for age, sex, physical health status parameters (underweight, anemia, smoking, and alcohol abuse), co-infections (HIV, helminths, viral, and bacterial pathogens), season, delay in diagnosis, non-TB medications, sequencing depth and sequencing batch; two-way interactions of CXR-findings or mycobacterial sputum burden with age, sex, physical health status parameters, co-infections and season were also evaluated. With the second model, we tested the marginal effect of clinical findings severity (as assessed by a TB score) while adjusting for delay in diagnosis, and the covariates included in the first model; except for BMI and anemia which are parameters used to compute the TB score. Interaction terms in both models were selected using stepwise-selection based on the Akaike information criterion (AIC), as implemented in the function *step* of the R package *stats*. On selected models, we tested normality of residuals, homoscedascity, non-multicolinearity, independence of errors and effect of influential observations. To assess the effect sizes of factors within selected models, we computed the partial eta squared statistic ($h_{p}^{2}$), which is the proportion of the Sum of Squares (SS) of the effect and the error that is attributable to the effect (Maher et al., 2013). *Post-hoc* tests included *t*-tests on adjusted means with R-package *emmeans* v1.4. The analysis is available at https://git.scicore.unibas.ch/TBRU/tbdarbiome_cases/-/blob/master/notebooks/12_MOT_alpha-diversity-and-clinical-features.ipynb.

Similarly, to investigate if compositional and structural changes of the microbial communities in the sputum of TB patients were associated with disease manifestations of pulmonary TB, we performed transformation-based redundant analysis (tb-RDA) with stepwise selection of interaction terms, and Permutational Analysis of Variance (PERMANOVA) as implemented in the R-package *vegan* v2.5. Response variables were CLR-transformed genus-level abundances. Continuous explanatory variables were always scaled. When comparing means among groups, *p-values* were adjusted for multiple comparisons following Holm-Bonferroni method. Analyses are available at https://git.scicore.unibas.ch/TBRU/tbdarbiome_cases/-/blob/master/notebooks/13_MOT_beta-diversity-and-clinical-features.ipynb.

## 3. Results

### 3.1. Cohort Characteristics

From November 2013 to November 2015, 663 new TB cases were enrolled at the Temeke district hospital in Dar es Salaam, Tanzania. Although it was originally planned to include all the sputa stored during these 2 years of patient enrollment, we did not have enough DNA extraction kits and ended up choosing a subset. We selected at random 4 9 × 9 boxes of sputa stored at –20°C; this resulted in 324 sputa. We still had reagents for another 10 DNA extractions, therefore we selected 10 more sputa from a randomly-picked 5th stored box. Thus, we included 334 (53%) sputa to investigate the potential relationship between sputum microbial composition and three aspects of TB-disease manifestations, at time of diagnosis, which include: (i) high mycobacterial load in sputum, (ii) severe clinical findings (signs and symptoms), and (iii) chest X-ray findings (see Figure 1). Sputum volumes were consistent across categories of TB-disease manifestations (Supplementary Figure S1).

In our study population and across categories of TB-disease manifestations, the distributions of demographics, physical health status, co-infections, non-TB medication, delay in diagnosis, season, and *MTB* genetic background are summarized in Table 1. Consistent with previous studies on this cohort (Mhimbira et al., 2017, 2019; Hella et al., 2018), most TB cases were males (72%), had anemia (74%), and were underweight (53%). A large proportion of TB cases were recruited during the *Dry* season (42%). Also, significant proportions were co-infected with HIV (24%), helminths (34%), viral (21%) and bacterial (34%) respiratory pathogens; 16% were smokers and 19% were alcohol abusers. Additionally, 69% reported a delay in TB diagnosis of more than 3 weeks, and 94% reported to have received non-TB medications before diagnosis.

**Table 1:** Characteristics of TB patients and associations with disease manifestations.

		**Mycobacterial load** ^****d****^			**Clinical findings** ^****e****^			**CXR findings** ^****f****^		
**Characteristics**	**Total**	**Low**	**High**	***p*-value^**a**^**	**Mild**	**Severe**	***p*-value^**a**^**	**Normal**	**Abnormal**	***p*-value^**a**^**
	**(*N* = 334)**	**(*N* = 127)**	**(*N* = 207)**		**(*N* = 223)**	**(*N* = 111)**		**(*N* = 58)**	**(*N* = 189)**	
**Demographics**										
**Sex**				0.660			0.559			0.646
Male	240 (71.86%)	89 (70.08%)	151 (72.95%)		163 (73.09%)	77 (69.37%)		38 (65.52%)	132 (69.84%)	
Female	94 (28.14%)	38 (29.92%)	56 (27.05%)		60 (26.91%)	34 (30.63%)		20 (34.48%)	57 (30.16%)	
**Age** (years)				0.434			0.159			0.742
Median (IQR)	33.00 (14.00)	33.00 (14.00)	33.00 (14.00)		34.00 (14.50)	31.00 (13.00)		33.00 (13.50)	33.00 (15.00)	
**Physical health parameters**										
**BMI** (kg/m2)				0.663			** <0.001**			0.586
Median (IQR)	18.26 (3.85)	18.27 (4.26)	18.26 (3.62)		19.49 (3.02)	16.07 (1.84)		19.09 (3.24)	18.22 (3.72)	
**Nutritional status**				0.788			** <0.001**			0.061
Normal weight	145 (43.41%)	56 (44.09%)	89 (43.00%)		137 (61.43%)	8 (7.21%)		34 (58.62%)	79 (41.80%)	
Underweight	176 (52.69%)	65 (51.18%)	111 (53.62%)		73 (32.74%)	103 (92.79%)		23 (39.66%)	100 (52.91%)	
Obesity	13 (3.89%)	6 (4.72%)	7 (3.38%)		13 (5.83%)	0 (0.00%)		1 (1.72%)	10 (5.29%)	
**Hemoglobin** (g/dL)				0.120			0.004			0.500
Median (IQR)	11.25 (2.92)	11.60 (3.30)	11.10 (2.90)		11.65 (2.80)	10.70 (3.10)		11.30 (3.45)	11.10 (2.70)	
Missing	18	7	11		15	3		7	6	
**Anemia status**				0.074			0.051			0.294
No	81 (25.63%)	38 (31.67%)	43 (21.94%)		61 (29.33%)	20 (18.52%)		16 (31.37%)	42 (22.95%)	
Yes	235 (74.37%)	82 (68.33%)	153 (78.06%)		147 (70.67%)	88 (81.48%)		35 (68.63%)	141 (77.05%)	
Missing	18	7	11		15	3		7	6	
**Respiration rate** (breaths/min)				0.157			** <0.001**			0.558
Median (IQR)	17.00 (5.00)	16.00 (4.00)	17.00 (5.00)		16.00 (4.00)	19.00 (8.00)		16.00 (5.00)	18.00 (4.00)	
**Smoker**				0.006			0.151			>0.999
no	280 (83.83%)	116 (91.34%)	164 (79.23%)		192 (86.10%)	88 (79.28%)		51 (87.93%)	165 (87.30%)	
yes	54 (16.17%)	11 (8.66%)	43 (20.77%)		31 (13.90%)	23 (20.72%)		7 (12.07%)	24 (12.70%)	
*Cigarettes per day*				0.545^†^			0.433^†^			>0.999^†^
Median (IQR)	7.00 (6.25)	6.00 (7.00)	8.00 (5.00)		8.00 (9.50)	7.00 (5.00)		6.00 (4.50)	6.50 (4.50)	
**Alcohol abuse**				0.230			>0.999			0.616
no	269 (80.54%)	107 (84.25%)	162 (78.26%)		180 (80.72%)	89 (80.18%)		49 (84.48%)	152 (80.42%)	
yes	65 (19.46%)	20 (15.75%)	45 (21.74%)		43 (19.28%)	22 (19.82%)		9 (15.52%)	37 (19.58%)	
**Co-infections**										
**HIV**				0.217			0.583			** <0.001**
Negative	244 (76.49%)	86 (72.27%)	158 (79.00%)		162 (75.35%)	82 (78.85%)		27 (50.94%)	150 (81.52%)	
Positive	75 (23.51%)	33 (27.73%)	42 (21.00%)		53 (24.65%)	22 (21.15%)		26 (49.06%)	34 (18.48%)	
Missing	15	8	7		8	7		5	5	
*CD4+ T cell counts* (cells/ul)				0.940			0.604			0.222
<200	22 (52.38%)	9 (56.25%)	13 (50.00%)		15 (48.39%)	7 (63.64%)		8 (44.44%)	12 (70.59%)	
>=200	20 (47.62%)	7 (43.75%)	13 (50.00%)		16 (51.61%)	4 (36.36%)		10 (55.56%)	5 (29.41%)	
*Previous ART*				0.914			0.776			>0.999
No	309 (94.50%)	116 (95.08%)	193 (94.15%)		204 (94.01%)	105 (95.45%)		51 (94.44%)	180 (95.24%)	
Yes	18 (5.50%)	6 (4.92%)	12 (5.85%)		13 (5.99%)	5 (4.55%)		3 (5.56%)	9 (4.76%)	
Missing	7	5	2		6	1		4	0	
**Respiratory viruses** ^b^				0.310			0.228			>0.999
Negative	227 (78.82%)	82 (75.23%)	145 (81.01%)		155 (81.15%)	72 (74.23%)		40 (76.92%)	124 (77.99%)	
Positive	61 (21.18%)	27 (24.77%)	34 (18.99%)		36 (18.85%)	25 (25.77%)		12 (23.08%)	35 (22.01%)	
Missing	46	18	28		32	14		6	30	
**Respiratory bacteria** ^b^				0.661			0.924			0.576
Negative	174 (66.16%)	66 (64.08%)	108 (67.50%)		112 (66.67%)	62 (65.26%)		30 (62.50%)	99 (68.28%)	
Positive	89 (33.84%)	37 (35.92%)	52 (32.50%)		56 (33.33%)	33 (34.74%)		18 (37.50%)	46 (31.72%)	
Missing	71	24	47		55	16		10	44	
**Helminths** ^c^				>0.999			0.503			0.772
Negative	222 (66.47%)	84 (66.14%)	138 (66.67%)		145 (65.02%)	77 (69.37%)		39 (67.24%)	133 (70.37%)	
Positive	112 (33.53%)	43 (33.86%)	69 (33.33%)		78 (34.98%)	34 (30.63%)		19 (32.76%)	56 (29.63%)	
**Season**				0.466			0.075			0.689
Short Rains (October–February)	120 (36.04%)	51 (40.16%)	69 (33.50%)		86 (38.74%)	34 (30.63%)		20 (34.48%)	75 (39.68%)	
Long Rains (March–May)	74 (22.22%)	26 (20.47%)	48 (23.30%)		53 (23.87%)	21 (18.92%)		14 (24.14%)	47 (24.87%)	
Dry (June–September)	139 (41.74%)	50 (39.37%)	89 (43.20%)		83 (37.39%)	56 (50.45%)		24 (41.38%)	67 (35.45%)	
Missing	1	0	1		1	0		0	0	
**MTBC lineage**				>0.999			0.960			0.096
L1	39 (17.33%)	14 (17.28%)	25 (17.36%)		25 (16.89%)	14 (18.18%)		6 (22.22%)	25 (17.48%)	
L2	8 (3.56%)	3 (3.70%)	5 (3.47%)		5 (3.38%)	3 (3.90%)		1 (3.70%)	5 (3.50%)	
L3	102 (45.33%)	37 (45.68%)	65 (45.14%)		69 (46.62%)	33 (42.86%)		6 (22.22%)	68 (47.55%)	
L4	76 (33.78%)	27 (33.33%)	49 (34.03%)		49 (33.11%)	27 (35.06%)		14 (51.85%)	45 (31.47%)	
Missing	109	46	63		75	34		31	46	
**TB Diagnostic delay** ^g^				>0.999			0.034			0.892
Delay < = 3 weeks	102 (30.54%)	39 (30.71%)	63 (30.43%)		77 (34.53%)	25 (22.52%)		19 (32.76%)	58 (30.69%)	
Delay >3 weeks	232 (69.46%)	88 (69.29%)	144 (69.57%)		146 (65.47%)	86 (77.48%)		39 (67.24%)	131 (69.31%)	
**Non-TB medication**				0.458			0.188			0.872
No	20 (5.99%)	10 (7.87%)	10 (4.83%)		17 (7.62%)	3 (2.70%)		4 (6.90%)	10 (5.29%)	
Penicillins	264 (79.04%)	100 (78.74%)	164 (79.23%)		172 (77.13%)	92 (82.88%)		46 (79.31%)	150 (79.37%)	
Other	50 (14.97%)	17 (13.39%)	33 (15.94%)		34 (15.25%)	16 (14.41%)		8 (13.79%)	29 (15.34%)	

a *Associations of categorical demographic and clinical characteristics with TB disease manifestations were assessed by chi-squared test. Associations with continuous variables were assessed by student t-tests or Wilcoxon rank-sum test (†) when normality could not be assumed. In bold, values below Bonferroni-adjusted significance criteria (α = 0.05/20);*

b *Anyplex^TM^ II RV16 and Allplex^TM^ Respiratory Panel 4, Seegene. 16 respiratory viruses and 6 bacterial species;*

c *8 helminth parasites: Ascaris lumbricoides, Enterobius vermicularis, hookworm, Hymenolepis diminuta, Schistosoma haematobium, Schistosoma mansoni, Strongyloides stercoralis and Trichuris trichiura;*

d *Mycobacterial load in the lungs was derived from Acid-Fast Bacilli (AFB) sputum smear grading (scanty, 1+, 2+, 3+) and categorized into ‘Low' (scanty or 1+) and ‘High' (2+ or 3+);*

e *To define clinical findings as mild or severe, we used an adapted TB score (0–12) (Mhimbira et al., 2017) that quantify the number of clinical findings observed in a patient during physical examination. As in Mhimbira and colleagues (Mhimbira et al., 2017), we defined clinical findings as severe if the TB score was ≥6; and as mild, otherwise;*

f *To screen for lung abnormalities, double readings of chest x-rays were performed by board-certified radiologists, and discrepancies were resolved by an independent reader;*

g *Duration of diagnostic delay was calculated based on the longest reported TB-related symptom and categorized into: “ ≤ 3 weeks” and “>3 weeks”; CXR, chest x-rays; IQR, interquartile range; ART, antiretroviral therapy*.

Pulmonary TB manifests in CXRs as parenchymal infiltrates/consolidations, cavities, pleural effusion, lymphadenopathy, and micronodules. As summarized in Table 2, in this cohort, parenchymal infiltrates were the most common (63%, *n* = 157), followed by cavities (36%, *n* = 89), pleural effusion (12%, *n* = 29), lymphadenopathy (10%, *n* = 25), and micronodules (5%, *n* = 13).

**Table 2:** Associations among TB-disease manifestations.

		**Mycobacterial load** ^****a****^			**Clinical findings** ^****b****^		
**Variable**	**Total**	**Low**	**High**	***p*-value^***c***^**	**Mild**	**Severe**	***p*-value^**c**^**
	**(*N* = 334)**	**(*N* = 127)**	**(*N* = 207)**		**(*N* = 223)**	**(*N* = 111)**	
**Mycobacterial load**				-			>0.999
Low	127 (38.02%)	127 (100.00%)	0 (0.00%)		85 (38.12%)	42 (37.84%)	
High	207 (61.98%)	0 (0.00%)	207 (100.00%)		138 (61.88%)	69 (62.16%)	
**Clinical findings**				>0.999			-
Mild	223 (66.77%)	85 (66.93%)	138 (66.67%)		223 (100.00%)	0 (0.00%)	
Severe	111 (33.23%)	42 (33.07%)	69 (33.33%)		0 (0.00%)	111 (100.00%)	
**CXR findings**				0.067			0.292
Normal	58 (23.48%)	29 (30.21%)	29 (19.21%)		43 (25.75%)	15 (18.75%)	
Abnormal	189 (76.52%)	67 (69.79%)	122 (80.79%)		124 (74.25%)	65 (81.25%)	
Missing	87	31	56		56	31	
**Lung infiltrates**				0.165			0.011
Absent	91 (36.84%)	41 (42.71%)	50 (33.11%)		71 (42.51%)	20 (25.00%)	
Present	156 (63.16%)	55 (57.29%)	101 (66.89%)		96 (57.49%)	60 (75.00%)	
Missing	87	31	56		56	31	
**Lung cavities**				0.313			0.023
Absent	159 (64.37%)	66 (68.75%)	93 (61.59%)		116 (69.46%)	43 (53.75%)	
Present	88 (35.63%)	30 (31.25%)	58 (38.41%)		51 (30.54%)	37 (46.25%)	
Missing	87	31	56		56	31	
**Pleural effusion**				0.926			0.706
Absent	218 (88.26%)	84 (87.50%)	134 (88.74%)		146 (87.43%)	72 (90.00%)	
Present	29 (11.74%)	12 (12.50%)	17 (11.26%)		21 (12.57%)	8 (10.00%)	
Missing	87	31	56		56	31	
**Lymphadenopathy**				0.925			0.527
Absent	222 (89.88%)	87 (90.62%)	135 (89.40%)		152 (91.02%)	70 (87.50%)	
Present	25 (10.12%)	9 (9.38%)	16 (10.60%)		15 (8.98%)	10 (12.50%)	
Missing	87	31	56		56	31	
**Micronodules**				0.044			>0.999
Absent	234 (94.74%)	87 (90.62%)	147 (97.35%)		158 (94.61%)	76 (95.00%)	
Present	13 (5.26%)	9 (9.38%)	4 (2.65%)		9 (5.39%)	4 (5.00%)	
Missing	87	31	56		56	31	

a *Mycobacterial load in the lungs was derived from Acid-Fast Bacilli (AFB) sputum smear grading (scanty, 1+, 2+, 3+) and categorized into “Low” (scanty or 1+) and “High” (2+ or 3+);*

b *To define clinical findings as mild or severe, we used an adapted TB score (0–12) (Mhimbira et al., 2017) that quantify the number of clinical findings observed in a patient during physical examination. As in Mhimbira and colleagues (Mhimbira et al., 2017), we defined clinical findings as severe if the TB score was ≥6; and as mild, otherwise;*

c *Associations of categorical demographic and clinical characteristics with TB disease manifestations were assessed by chi-squared test. Associations with continuous variables were assessed by student t-tests or Wilcoxon rank-sum test (†) when normality could not be assumed. In bold, values below Bonferroni-adjusted significance criteria (α = 0.05/7)*.

#### 3.1.1. Factors Associated With Manifestations of Active Pulmonary TB

After Bonferroni adjustment of significance level (0.05) for multiple comparisons, we observed a few associations with any of the three aspects of disease manifestations investigated in this study (see Table 1): i) HIV with normal CXRs (*p* < 0.001; Chi-squared test), 49% of patients with normal CXRs had HIV as compared to only 18% of those with abnormal CXRs; and ii) underweight nutritional status and higher respiratory rate with severe clinical findings, as compared to patients with mild clinical findings. These associations are expected given that these factors were clinical parameters considered in the *TB score* used to grade the severity of clinical findings.

#### 3.1.2. Associations Among Manifestations of Active Pulmonary TB

We also investigated if clinical manifestations were associated with each other (see Table 2). In this cohort, and at a Bonferroni-adjusted significance level of 0.05, severe clinical findings for TB were neither associated with high mycobacterial load in sputum nor with abnormal CXRs. In addition, abnormal CXRs were not associated with high mycobacterial load. However, when looking at specific types of CXR findings, the presence of lung cavities was associated with lower BMI (*p* < 0.001; *t*-test; see Supplementary Table S1). Additionally, there was a significant effect for sex (*p* = 0.002; Chi-squared test) on the presence of parenchymal infiltrates, 76% of patients with lung infiltrates were males as compared to 56% of those without lung infiltrates detected on chest X-rays (see Supplementary Table S1).

### 3.2. Description of the Sequencing Data

We analyzed the total DNA of all sputum samples (334 patients) by 16S rRNA gene amplicon sequencing (16S-AS). We obtained about 24.6 million reads, median reads per sample was 55,672 (IQR: 36,666–98,143). After filtering, denoising and chimera removal with DADA2, about 12.4 million sequences remained; 28,231 (IQR: 19,227–45,916) median reads per sample. Samples had a median of 171 (IQR:111–252) amplicon sequence variants (ASVs). Additional filtering removed 8 ASVs flagged as potential contaminants by Decontam, as described in section Identification of Potential Contaminants (Supplementary Figure S2), and 27 observations which had <12,000 reads (threshold at which most samples approached plateau in richness rarefaction curves; Supplementary Figure S3), which resulted in 307 16S-AS abundance profiles.

Based on DNA concentration and quality (limited fragmentation, see methods), 125 samples were selected for Whole-Metagenome Shotgun sequencing (WMS-S). We obtained about 3.96 × 10^9^ reads, median reads per sample was 28,948,058 (IQR: 22,116,534–37,234,300), and the percentage of reads annotated as human ranged from 2.6 to 92.1. After filtering out human and low-quality reads, about 1.11 × 10^9^ reads remained; median reads per sample was 4,836,788 (IQR: 3,366,538–7,818,888). Additional filtering removed five species identified as contaminants (Supplementary Figure S4) by Decontam, three species not expected as part of a mock community (Supplementary Figure S5), 19 samples with more than 20% reads assigned to contaminant species, two samples with 100% of unclassified reads, and 15 samples with <50% of reads assigned to human DNA; thus, 89 WMS-S abundance profiles were included in further analyses.

**Figure 2:**
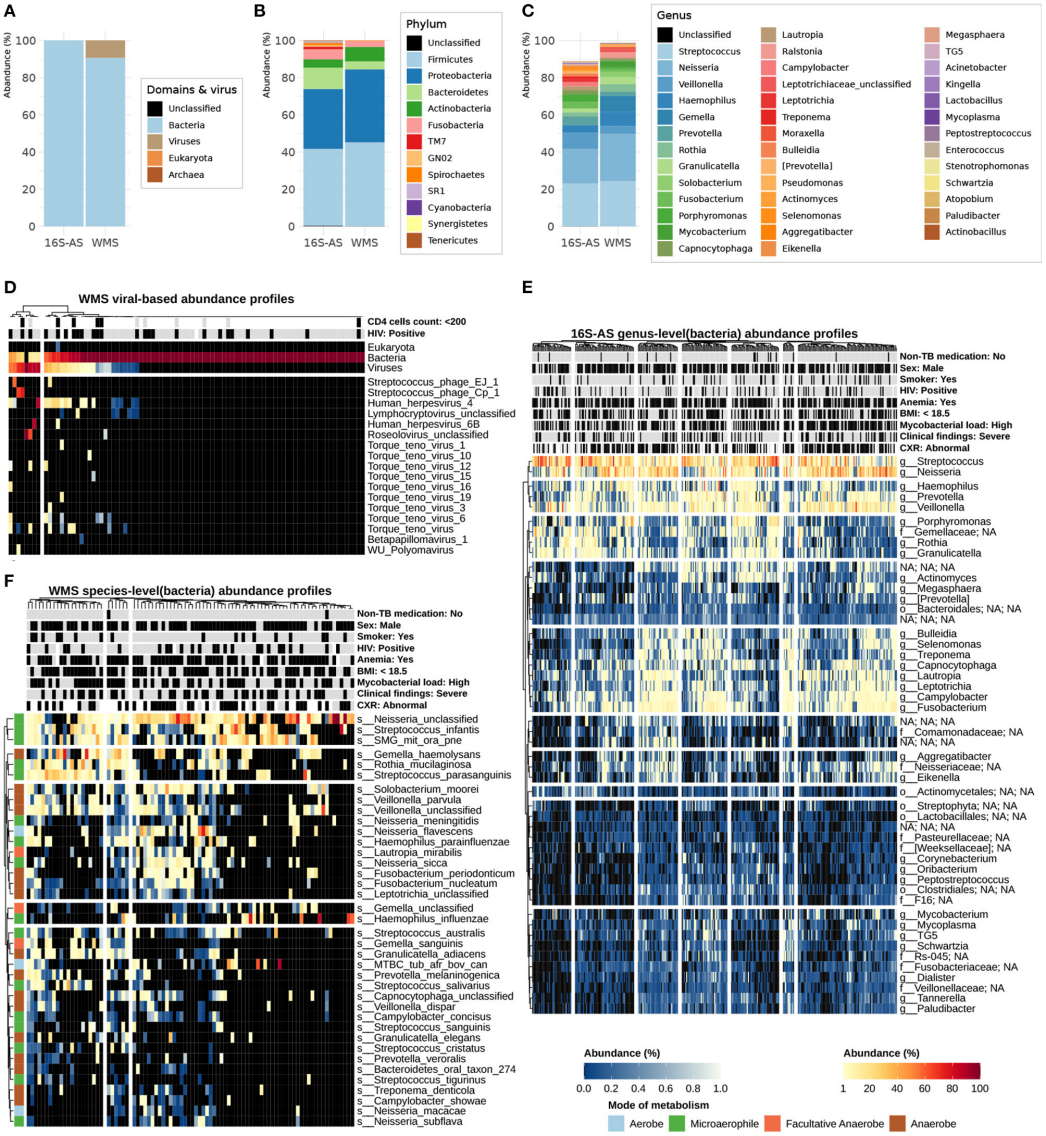
Detailed overview of the sputum microbial composition in TB patients. Per sequencing dataset, mean relative abundances (%) at the domain (including viruses) level **(A)**, bacterial-phylum level **(B)**, and bacterial-genus level which includes genera with at least 0.1% relative abundance **(C)**; in the legends, below "Unclassified", taxa are arranged from high to low abundance. Heatmap of relative abundances (%) for domains and viruses **(D)**; viral composition is break down into species-level components; columns (sputum samples, *N* = 89) were annotated with the HIV status of the TB patient; taxonomic profiles were derived from WMS-S data. Bacterial composition is break down into species- and genus-level components; species-level abundance profiles were derived from WMS-S data and genus-level abundance profiles were derived from 16S-AS data. Heatmap of bacterial genus-level abundance profiles **(E)** displays genera detected in at least 50% of sputum samples while heatmap of bacterial species-level abundance profiles **(F)** displays species detected in at least 15% of sputum samples. Sputum samples and bacterial species/genera are sorted according to hierarchical clustering using Aitchison distances (for additional details, see methods). For the heatmap of bacterial species-level abundance profiles, bacterial species are annotated by reported mode of metabolism. Sputum samples are annotated by different aspects of TB disease manifestations (sputum mycobacterial load, chest X-ray, and clinical findings) and linked demographic and clinical characteristics; annotation labels (variable:level) include the variable's name and its level displayed in black in the annotation colors, which are gray for alternative levels and white for missing values.

16S-AS and WMS-S provided complementary snapshots of the microbial composition in the sputum of TB patients (see Figure 2). Although 16S-AS was able to profile a larger number of samples, species-level resolution was limited; most (86%) of the ASVs detected by DADA2 did not have a species classification. WMS-S on the other hand, provided a cross-domain and species-level resolution, evidenced by the detection of viruses and fungi, and by the accurate taxonomic profiling of a mock community composed of even abundances of 20 species (ATCC® MSA-1002^TM^; Supplementary Figure S5A). However, WMS-S resolution came at the cost of losing detection of low abundant taxa; WMS-S detected 78 bacterial genera while 16S-AS detected 319 genera, and genera not-detected by WMS-S had mean relative abundances generally below 0.01% (Supplementary Figure S6).

Based on these observations, we decided to use the 16S-AS dataset for genus-level analysis as this dataset included more samples and captured 2.5 times more genera than the WMS-S dataset. Considering the limited species-level accuracy in our 16S-AS and the accurate taxonomic profiling of a mock community by our WMS-S processing pipeline, we used the WMS-S dataset for species-level analysis.

### 3.3. Microbial Composition of Sputum in TB Patients

Taxonomic assignments derived from 89 WMS abundance profiles showed that microbial communities in the sputum of TB patients were primarily composed of bacteria (90.6, 83.7–97%; mean, 95% CI) and viruses (9.4, 5.6–19.6%; mean, 95% CI) (Figure 2A). The presence of members of the Eukaryota or Archaea domains was rather rare, or below detection limit: only one sample, among those screened by WMS-S, had reads assigned to *Candida albicans* and four samples, among those screened by 16S-AS, had *Metanobrevibacter* ASVs.

Taxonomic assignments derived from 307 16S-AS abundance profiles showed that the bacterial content of the sputum in TB patients was dominated by 10 phyla (Figure 2B): *Firmicutes, Proteobacteria, Bacteroidetes, Fusobacteria, Actinobacteria, TM7* (*Saccharibacteria*), *Spirochaetes, GN02* (*Gracilibacteria*), *SR1* (*Absconditabacteria*), *Tenericutes*; together they accounted for 99.3% of the total bacterial relative abundance measured by 16S-AS. At the genus level (Figure 2C), 16 genera which had mean relative abundances of at least 1% covered approximately 82% of the total bacterial relative abundance. In agreement with previous studies (Krishna et al., 2016; Hong et al., 2018; Sala et al., 2020), the top 10 most abundant genera included: *Streptococcus, Neisseria, Veillonella, Prevotella, Haemophilus, Porphyromonas, Fusobacterium, Campylobacter, Rothia*, and *Lautropia*.

#### 3.3.1. Limited Detection of *MTB*

As in previous studies, the relative abundance of putative *MTB* in the airways microbial communities of TB patients was low. One *Mycobacterium* ASV was detected in 52% of the 16S-AS profiles, median relative abundance per sample was 0.01% (IQR:0.0–0.14%). However, this ASV was classified as *Mycobacterium gordonae*. ASVs were determined using only the first reads from our 16S-AS paired-end dataset which reduced the length of our fragments and therefore the capability to discriminate closely related species. Thus, QIIME2's taxonomic classifier might be incorrectly assigning *MTB* ASVs to species *M. gordonae*. Conversely, MetaPhlAn2 detected the *MTB* complex in 30% of the WMS-S profiles, median relative abundance per sample was 0.0% (IQR: 0.0–0.04%). The relative abundances of neither the putative *Mycobacterium* ASV nor the species identified as part of the *MTB* complex were correlated with the sputum Mycobacterial load, as assessed by AFB smear grading (Supplementary Figures S7, S8).

### 3.4. Microbial Associations Linked to Diversity and Structuring of Sputum Microbial Assemblages in TB Patients

#### 3.4.1. Inverse Relationships Between *Streptococcus* and Anaerobes

To have an initial overview of the microbial composition in the sputum of TB patients and of the underlying structuring of those communities, we created heat-maps to visualize the relative abundances for genera and species across all samples (Figures 2E,F). Since we found high correlations between ordinations based on Aitchison distances of rarefied and non-rarefied abundances (Procrustes *R*^2^ = 0.98, *p* = 0.001; Supplementary Figure S9), samples and taxa were sorted according to hierarchical clustering using Aitchison distances computed on non-rarefied taxonomic profiles.

**Figure 3:**
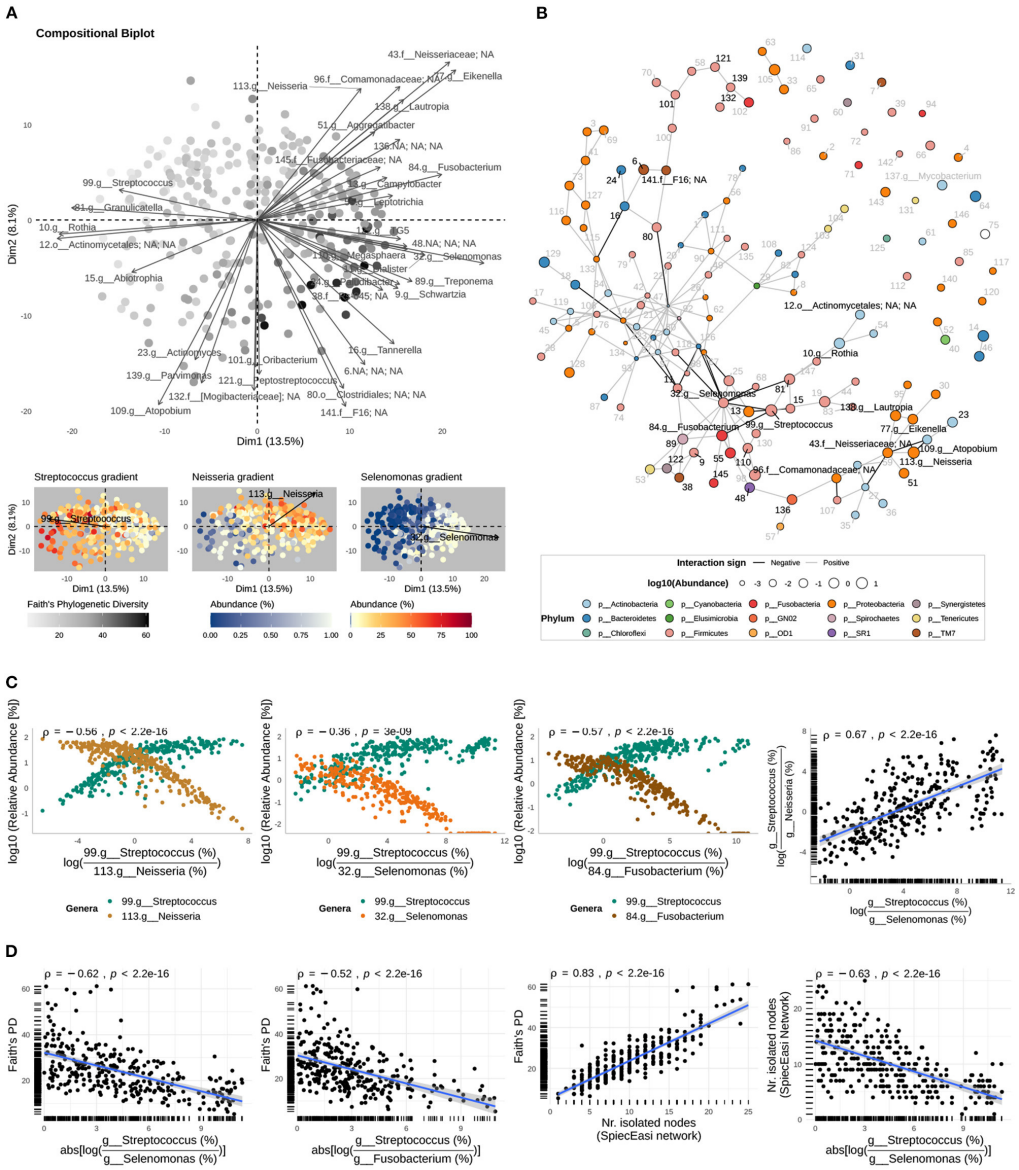
Genus-level community interactions. **(A)** Relative variation biplot of genus-level community compositions (centered log-ratio transformed) shows the relative variation of the log-ratios for the top 35 taxa (arrows, taxa best represented by the axes of the biplot), and sputum samples (dots); the length of the imaginary line connecting the tips of two arrows, representing two taxa, is proportional to the variation of the log-ratio of the corresponding abundances; sputum samples are colored by the Faith's phylogenetic diversity, and the relative abundances of genera *Streptococcus, Neisseria*, and *Selenomonas*. **(B)** Interaction network inferred with the SParse InversE Covariance Estimation for Ecological ASociation Inference (SPIEC-EASI) method where nodes represent genera, colored by the corresponding phylum, and of sizes corresponding to the relative abundance (%) averaged across sputum microbial assemblages and in logarithmic scale; we display the labels of the top 10 taxa best represented by the relative variation biplot (See Supplementary Figure S8 for the complete set of labels). **(C)** The relative abundances of *Streptococcus* and genera negatively interacting with it as function of the abundance log-ratio between *Streptococcus* and the genus interacting with it; Spearman's rank-order correlation coefficient (ρ) between the relative abundances (log10) are shown. The relationship between the Streptococcus-to-Neisseria and Streptococcus-to-Selenomonas log-ratios is also shown. **(D)** Faith's PD as a function of the strength of the interactions between *Streptococcus* and the anaerobes *Selenomonas* and *Fusobacterium*, and as a function of the number of isolated nodes in the interaction network. In addition, the relationship between the latter and the strength of the interaction between *Streptococcus* and *Selenomonas* is shown. Monotonic relationships were assessed by Spearman's rank-order correlation (ρ).

Although preliminary visualizations with heat-maps did not show clear-cut clustering of the microbial communities, they showed a trade-off between genera *Streptococcus* and *Neisseria*; as the abundance of *Streptococcus* decreased, *Neisseria* increased (Figure 2E). This relationship was further supported by compositional biplots where vectors representing variation of these two genera, relative to the geometric mean of the other genera within a sample, pointed in different directions (Figure 3A); moreover, the relative abundance (log10) of both taxa were negatively correlated (Spearman ρ = −0.56, *p* < 0.001; Figure 3C).

In compositional biplots, the length of the line (i.e., link) connecting the tips of two vectors representing two taxa is proportional to the variation of the corresponding log-ratio of their abundances. In this sense, the genus-level compositional biplot suggests that genus *Streptococcus* also has an inverse relationship with genus *Selenomonas* and that their log-ratio is even more variable than the one between *Streptococcus* and *Neisseria* (Figure 3A). However, these qualitative observations have to be interpreted carefully because the axis of the biplot only explained 21.6 % of the total variation in community composition. Thus, we plotted the relative abundances of these genera, which confirmed their inverse relationship (Figure 3C and Supplementary Figure S10B).

Compositional biplots suggest that there are other associations among taxa abundances in the sputum of TB patients, including strong positive correlations (i.e., constant ratios across sputum samples). However, as mentioned earlier, observations based on our compositional biplots were limited by the quality of their projections (small proportion of variance explained). To have a better representation and inference of associations among taxa, we applied the SParse InveresE Covariance Estimation for Ecological ASociation Inference (SPIEC-EASI) to infer genus- and species-level interaction networks. At the genus level, the inferred network confirmed the inverse relationship of *Streptococcus* with *Selenomonas* (Figure 3B and Supplementary Figures S11, S12). However, no direct interaction between *Streptococcus* and *Neisseria* was inferred. Instead, the reconstructed network suggested an indirect negative relationship between *Streptococcus* and *Neisseria* as a result of cascading effects in the network, triggered by negative interactions of *Streptococcus* with anaerobic genera *Selenomonas* and *Fusobacterium* (Figure 3B and Supplementary Figure S12); an observation supported by the positive correlation between the *Streptococcus*-to-*Neisseria* and the *Streptococcus*-to-*Selenomonas* log-ratios (Spearman ρ = 0.67, *p* < 0.001; Figure 3C right). These microbial associations were also confirmed by the SparCC approach (Supplementary Figure S13). To simultaneously visualize the relationships among the relative abundance of the four genera (*Streptococcus, Neisseria, Selenomonas*, and *Fusobacterium*), we created bar plots per sample with the relative abundance of the four genera (Supplementary Figure S14). Together, these observations indicate that the inverse relationships between *Streptococcus* and anaerobes *Selenomonas* and *Fusobacterium* structure the TB-associated sputum microbial communities in the following way: at one extreme of the spectrum there is dominance of Streptococcus while at the other extreme there is an elevated content of *Neisseria, Fusobacterium*, and *Selenomonas*, but *Streptococcus* is depleted. This spectrum includes intermediate states with a relative balance between these taxa (Figure 3A bottom and Supplementary Figure S14).

Although we observed an inverse relationship at the species-level between *Neisseria sicca* and *Streptococcus parasanguinis* (Supplementary Figure S15 left), the species-level network reconstructed using the WMS-S dataset did no show a direct interaction between these two species (Supplementary Figures S15, S16). Three *Selenomona spp*. (*S. flueggei, S. noxia*, and *S. sputigena*) were detected in the WMS-S dataset, however, they were detected in only three samples or less (Supplementary Figure S17). The limited detection of *Selenomonas spp*. in the WMS-S dataset is expected considering the low relative abundance of *Selenomonas* (mean = 1.05% *SD* = 1.9; 16S-AS dataset).

#### 3.4.2. The Strength of Interactions Between *Streptococcus* and Anaerobes Impacts Negatively Phylogenetic Diversity

Multiple studies of ecological communities have shown that the strength of interactions between members of a community can determine the structure and diversity of the community (McCann et al., 1998; Ratzke et al., 2020). Indeed, We observed in the genus-level compositional biplot a gradient of phylogenetic diversity that decreases in the direction of Streptococcus but increases in the direction of Selenomonas and other anaerobes (Figure 3A). Thus, we hypothesized that the strength of the network-based interactions of *Streptococcus* with *Selenomonas* or *Fusobacterium* within individual assemblages might have an impact on diversity. Consequently, for each sputum sample, we measured the strength of the interaction between these genera as the absolute value of the log-ratio of their abundances. Large values indicate large differences in their relative abundance and therefore large negative effects of one taxon on the other, while small values indicate co-occurrence and therefore weak mutual exclusion. To measure diversity within individual microbial assemblages, we computed Faith's phylogenetic diversity (PD). We found negative correlations of PD with the strength of the *Streptococcus*-*Selenomonas* interaction (Spearman ρ = −0.62, *p* < 0.001) and with the strength of the *Streptococcus*-*Fusobacterium* interaction (Spearman ρ = −0.52, *p* < 0.001). Consistent with a recent study by Ratzke et al. (2020), this result suggests that strong negative interactions in sputum microbial assemblages impact negatively the diversity of the community (Figure 3D).

Both the SPIEC-EASI and the SparCC genus-level interaction networks, derived from the 16S-AS dataset, also revealed a group of isolated nodes in the networks (Figure 3B and Supplementary Figure S18A) whose accumulation in sputum samples was positively correlated with Faith's PD (Spearman ρ = 0.83, *p* < 0.001, SPIEC-EASI network; Spearman ρ = 0.85, *p* < 0.001, SparCC network; Figure 3D and Supplementary Figures S18B,C left). These isolated nodes represent genera whose presence was not consistent across all sputum samples or whose abundance was not associated with the abundance of other genera. In decreasing order of mean relative abundance, these genera included: *Porphyromonas, Mycobacterium, Moraxella, Lactobacillus, Corynebacterium, Cardiobacterium, Pyramidobacter, Lachnoanaerobaculum, Pasteurela, Filifactor, Butyrivibrio, Helicobacter, Streptobacillus*, putative genus *SHD-231, Johnsonella*, and *Peptococcus*. These genera belongs to different phyla, which supports their effect on increasing Faith's PD when they accumulate within a sputum sample.

Consistent with our finding that Faith's PD was negatively correlated with the strength of the *Streptococcus*-*Selenomonas* interaction, we also found a negative correlation between the latter and the accumulation of these potentially transient genera (Spearman ρ = −0.63, *p* < 0.001, SPIEC-EASI network; Spearman ρ = −0.59, *p* < 0.001, SparCC network; Figure 3D and Supplementary Figures S18B,C right).

Taken together, these findings suggest a key role of interactions between *Streptococcus* and certain anaerobes in shaping the composition and structure of the microbial communities in the sputum of TB patients.

### 3.5. Microbial Diversity and Composition of Sputum and Manifestations of Pulmonary TB

#### 3.5.1. Underweight Status Determines the Association Between Faith's PD and Abnormal CXRs

**Figure 4:**
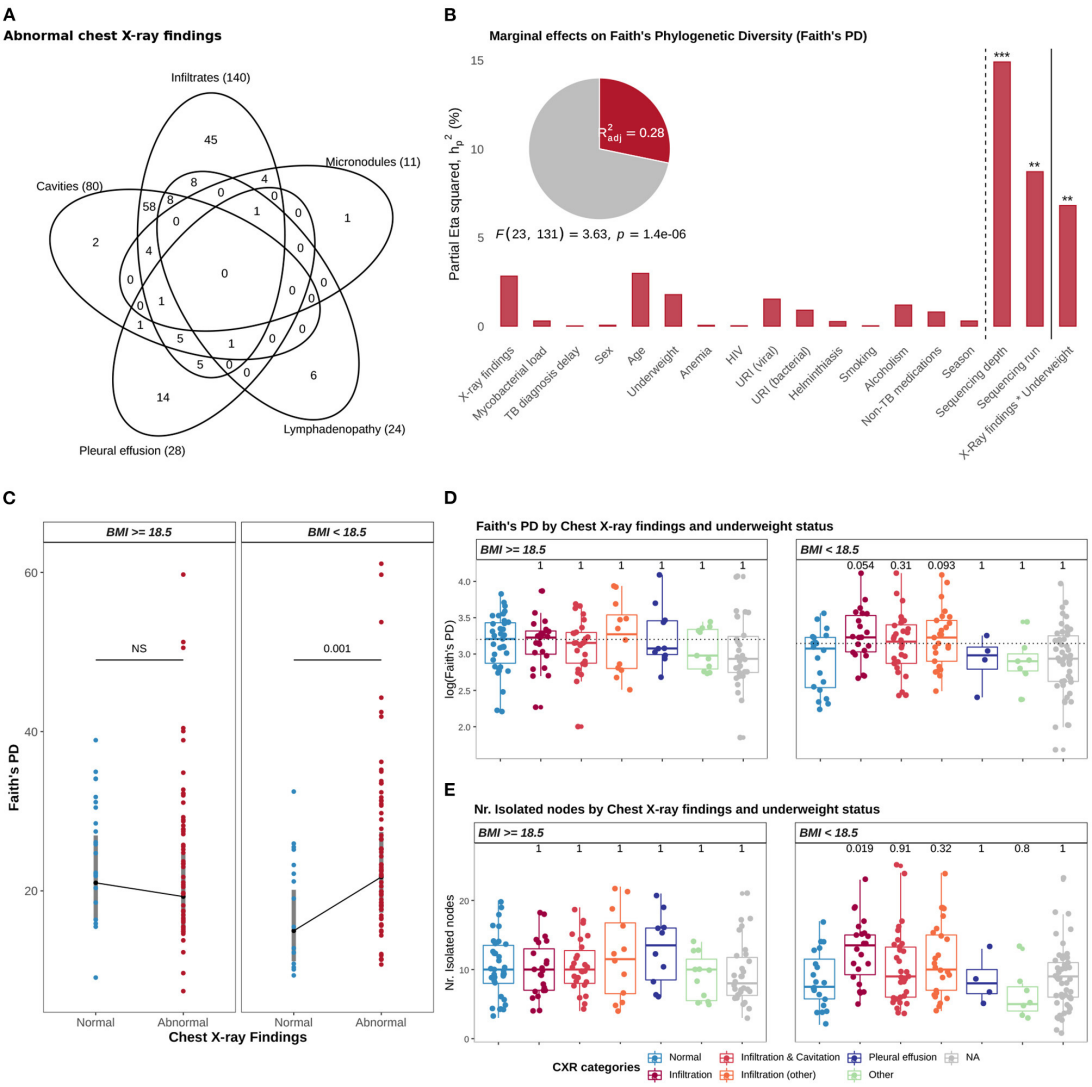
Associations with Faith's phylogenetic diversity (PD). **(A)** Venn diagram showing the distribution of TB patients across the five types of lesions comprising abnormal CXRs findings. **(B)** The graph illustrates a multi-way ANCOVA model testing for associations with Faith's PD (log10); the height of the bars correspond to the proportion of Faith's PD variance accounted by variables included in the model (marginal effect sizes), and measured by the partial Eta squared statistic ($h_{p}^{2}$, see methods); the adjusted R-squared statistic, the significance of the model and of the variables are shown: *p ≤ 0.05, **p ≤ 0.01, ***p ≤ 0.001, and ****p ≤ 0.0001. **(C)** Plot showing the estimated marginal means of Faith's PD by Chest x-ray findings and underweight status, based on the ANCOVA model showed in B; gray bars correspond to 95% confidence intervals of the estimated means. **(D)** Distribution of Faith's PD (log10), by CXR categories and underweight status; Student *t*-tests were applied for pairwise comparison of means (reference: “Normal” CXRs). **(E)** Distribution of the number of isolated nodes, from the genus-level interaction network, by CXR categories and underweight status; Wilcoxon rank-sum tests were applied for pairwise comparison of means (reference: “Normal” CXRs). In **(D,E)**, CXR categories were defined as “Normal” (none of the five CXR signs), “Infiltration” (only parenchymal infiltrates observed in CXRs), “Infiltration and Cavitation” (infiltrates with cavities, but no additional CXR signs), “Infiltration (other)” (infiltrates with any other CXR sign, accompanied or not by cavities), “Pleural effusion” (no additional CXR signs) and “Other” (patients without parenchymal lesions and one patient with pleural effusion and cavities). In **(D,E)**, *p*-values were adjusted for multiple comparisons with Holm-Bonferroni Method.

Abnormal CXRs in this cohort comprised five types of CXR signs: parenchymal lesions (infiltrates/consolidations), cavities, pleural effusion, lymphadenopathy, and micronodules. Parenchymal lesions were the most frequent type (63.3%, *n* = 157) of CXR signs in this cohort and were present alone (32%, *n* = 50) or together with the other CXR signs (Figure 4A). For instance, 78 out of the 80 (97.5%) patients with lung cavities, included in the ANCOVA models, had also parenchymal lesions. Although variation of Faith's PD was neither associated with high mycobacterial load in the sputum of TB patients nor with severe clinical findings, multi-way ANCOVA models (see methods) revealed that underweight status (BMI <18.5) determined an association between Faith's PD and abnormal CXR findings (*p* = 0.002, ANCOVA; Figure 4B). More specifically, differences in the estimated marginal means of Faith's PD between TB patients with abnormal CXRs and those with normal CXRs were significant in underweight TB cases (*p* = 0.001, *t*-test) but not in those with BMI above 18.5 (Figure 4C). In a separate ANCOVA model that included the genetic background of *MTB*, neither MTB genetic background nor the interaction Underweight status * CXR findings had significant effects on Faith's PD (Supplementary Figure S13), potentially as a consequence of considerable reduction in degrees of freedom (Total *df* = 103).

To further characterize the association of abnormal CXRs with Faith's PD, among underweight TB patients, we re-categorized abnormal CXRs into 5 groups: i) parenchymal lesions (only), no additional CXR signs; ii) parenchymal lesions with cavities but no additional CXR signs; iii) parenchymal lesions and other, where 'other' refers to any additional CXR sign accompanied or not by cavities; iv) pleural effusion (only), no additional lesions; and v) other, which aggregates patients without parenchymal lesions and one patient with pleural effusion and cavities. We found that among underweight TB cases, the association of abnormal CXRs with Faith's PD was mediated by increased levels of Faith's PD in TB patients with lung parenchymal infiltrates, in comparison to those with normal CXRs (*p*_*adj*_ = 0.05, *t*-test; Figure 4D). Since we observed that Faith's PD was positively correlated with an accumulation of potentially transient genera (i.e., isolated nodes in the genus-level interaction network, Figure 3D), we, therefore expected higher levels of transient genera in underweight TB patients with lung parenchymal infiltrates, in comparison to underweight TB patients with normal CXRs. A Wilcoxon rank-sum test confirmed our expectation (*p*_*adj*_ = 0.019; Figure 4E). These observations suggest that accumulation of transient genera potentially drives the increased levels of Faith's PD in the sputum microbial community of underweight TB patients with lung parenchymal infiltrates.

#### 3.5.2. Underweight Status Determines the Association Between Compositional Variation and CXRs Findings

**Figure 5:**
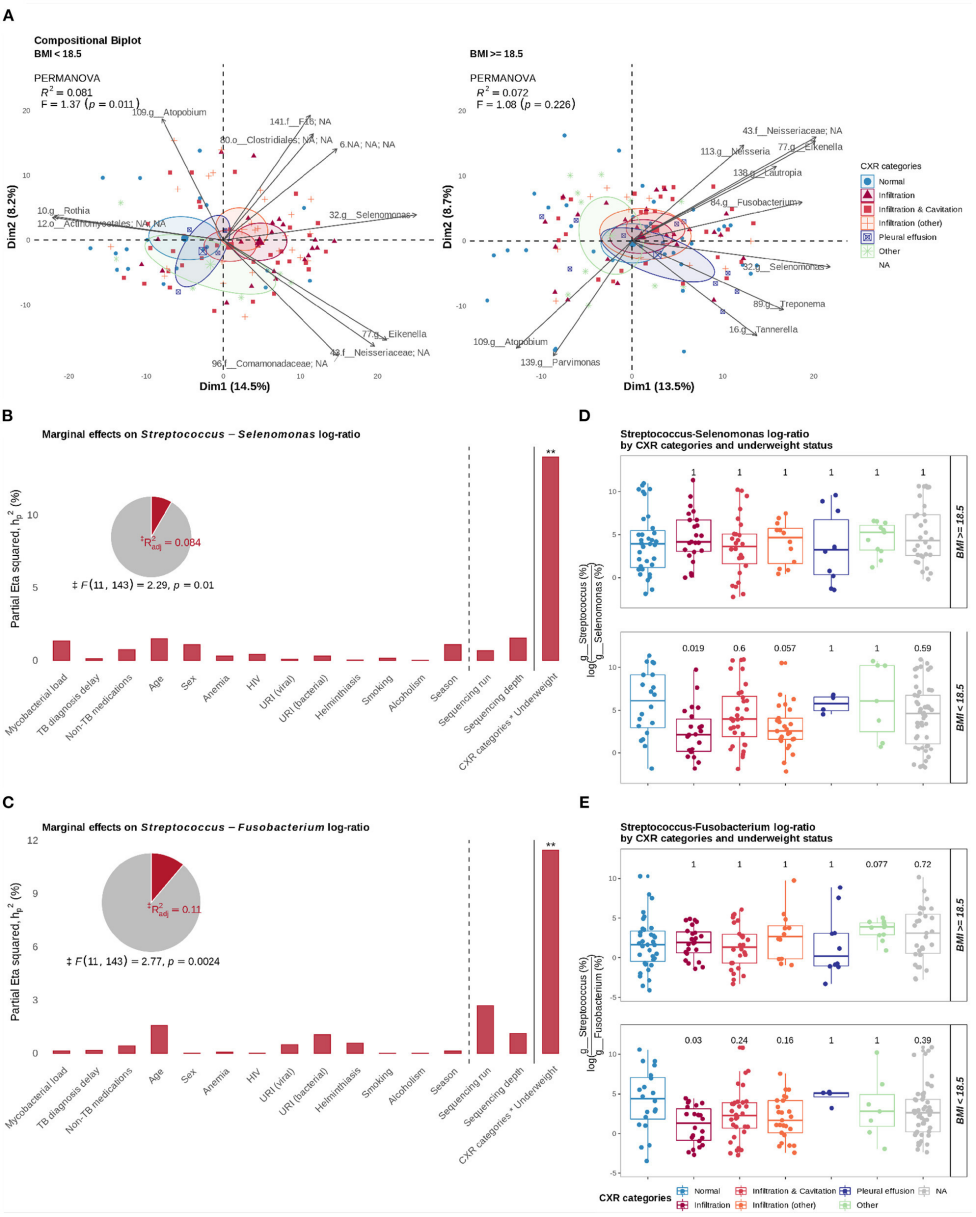
Associations with compositional variation and log-ratios of interacting taxa. **(A)** Relative variation biplots, stratified by underweight status (BMI < 18.5 and BMI ≥ 18.5), show the effect of underweight status on the association between differences in genus-level log-ratio abundances and CXRs categories. **(B)** Marginal effect sizes (partial eta squared) of variables included in a PERMANOVA model testing associations with the *Streptococcus*-to-*Selenomonas* log-ratio. **(C)** Marginal effect sizes of variables included in a PERMANOVA model testing associations with the *Streptococcus*-to-*Fusobacterium* log-ratio. **(D)** Distribution of the *Streptococcus*-to-*Selenomonas* log-ratio by CXR categories and underweight status. **(E)** Distribution of the *Streptococcus*-to-*Fusobacterium* log-ratio by CXR categories and underweight status. In **(B,C)**, the significance of the variables are shown: *p ≤ 0.05, **p ≤ 0.01, ***p ≤ 0.001, and ****p ≤ 0.0001; ‡ The F-statistic and the p-value of the model correspond to a model considering only significant terms. In **(D,E)**, Wilcoxon rank-sum tests were applied for pairwise comparison of means (reference: “Normal” CXRs); shown *p*-values were adjusted for multiple comparisons with Holm-Bonferroni method.

Following up on the observed associations with Faith's PD, we also evaluated if inter-patient variation (β diversity) of genus-level abundance profiles (CLR-transformed) was associated with CXR findings categorized by the type of lesions. Using transformation-based redundancy analysis (tb-RDA), step-forward selection of two-way interactions, and PERMANOVA, we also found that underweight status determined an association between compositional variation and CXR findings (*p* = 0.007, PERMANOVA; adjusted for age, sex, physical health status parameters, co-infections, season, mycobacterial load, delay in diagnosis, non-TB medications, sequencing depth, and sequencing batch). As it was observed for Faith's PD, differences in genus-level log-ratio abundances were associated with CXRs findings in underweight TB cases (*p* = 0.01, PERMANOVA; adjusted for age, sex, mycobacterial load, delay in diagnosis, HIV, and sequencing depth) but not in those with BMI above 18.5 (Figure 5A). As expected, this association was mediated by the *Streptoccocus*-to-*Selenomonas* log-ratio (*p* = 0.001, PERMANOVA; Figure 5B) and the *Streptoccocus*-to-*Fusobacterium* log-ratio (*p* = 0.01, PERMANOVA, Figure 5C). More specifically, we found that only among underweight TB cases, and in comparison to those with normal CXRs, TB patients with parenchymal infiltrates showed a significant decrease of both the *Streptoccocus*-to-*Selenomonas* log-ratio (*p*_*adj*_ = 0.02, Wilcoxon rank-sum test; Figure 5D) and the *Streptoccocus*-to-*Fusobacterium* log-ratio (*p*_*adj*_ = 0.03, Wilcoxon rank-sum test; Figure 5E).

Together, our results suggest that in underweight TB patients with lung parenchymal infiltrates, a change of the local lung environment is associated with a shift in the composition and diversity of the sputum microbiome. An accumulation of transient genera and an increase in the abundance of anaerobic genera *Selenomonas* and *Fusobacterium* with depletion of genus *Streptococcus* potentially mediated this shift.

### 3.6. Presence of Opportunistic Viruses in Microbial Assemblages Linked to HIV Co-infection

DNA Viruses were detected in 37% of the samples screened by WMS-S; the most common viruses were Human Herpesvirus 4 (HHV-4, also known as Epstein-Barr virus) and Torque Teno Virus (TTN) (Figure 2D). A few sputum samples (*n* = 7) had excessive viral relative abundance (> 51 %, equivalent to *mean*+2*SD*), dominated by one or few species (Streptococcus phages, HHV-4, and unclassified Roseolovirus). Although, both HHV-4 and TTN viruses are generally carried asymptomatically, these viruses can expand or cause (respiratory) infections, specially in immuno-compromised individuals (Thom and Petrik, 2007; Reid et al., 2016). Thus, we investigated if the detection of viruses in the sputum of TB patients, which in this cohort is dominated by both HHV-4 and TTN viruses, was linked to HIV. The proportion of HIV in TB patients with detectable viral DNA in their sputa was 43%, as compared to 21% of TB patients with no viral DNA detected in their sputa. However, a chi-squared test of independence showed a borderline significant association between the two variables (*p* = 0.06).

To look for associations between the identified viruses and bacterial species, we included virus abundances when reconstructing the network of microbial associations with SpiecEasi (Supplementary Figure S21A). SpiecEasi inferred direct positive associations between TTVs, *Moraxella catarrhalis* and *Streptococcus vestibularis* (Supplementary Figure S21A). However, we have to be skeptical about these associations because they were derived from the detection of both *M. catarrhalis* and *S. vestibularis* in few sputum samples (*n* = 3), see Supplementary Figure S21B.

Although our observations of the viral fraction of the microbial communities in the sputum of TB patients are exploratory, they highlight the overlooked importance of the virome in the airways of TB patients co-infected with HIV.

## 4. Discussion

Patients with pulmonary TB show remarkable disease heterogeneity (Lin and Flynn, 2018). Likewise, inter-individual variability of the microbial communities in the airways of TB patients is also large (Zhou et al., 2015; Hong et al., 2016). Whether those two sources of variation among TB patients are related should be explored in large human cohorts, where potential confounding factors can also be considered. Here, we present such a study. In a large human cohort of patients with pulmonary TB, stratified by three types of disease manifestations, we first assessed the distribution of potential confounding factors: demographics, physical/nutritional status, co-infections, and previous medication intake. We then characterized the diversity, composition, and structure of the microbial communities in the sputa. And finally, we assessed if the overall variability in diversity and community structure could be explained by differences in disease manifestations or by other co-factors or interactions with them.

As previously reported for the human microbiome and more recently for the airway microbiome (Faust et al., 2012; Einarsson et al., 2019), we found numerous associations among members of microbial communities in the respiratory tract of TB patients. However, we are reporting for the first time key microbial associations that were involved in shaping the structure of microbial assemblages found in the sputa of patients with pulmonary TB. In particular, our results showed that negative associations of genus *Streptococcus* with *Selenomonas* and *Fusobacterium* were important drivers of diversity and compositional variation; sputum microbial communities could be separated into groups where one taxa dominated over the other. This finding is not entirely surprising, considering well known phylum-level trade-offs (e.g., *Bacteroidetes*/*Firmicutes*) described for the gut microbiome in relation to obesity (Turnbaugh et al., 2009). Interestingly and in agreement with a recent study (Ratzke et al., 2020), the *Streptococcus*-to-*Selenomonas/Fusobacterium* trade-offs were also important drivers of Faith's PD. For instance, PD reached highest levels when *Streptococcus* and *Selenomonas* co-existed at equal proportions (approximately 1% relative abundance for both taxa, and dropped as *Selenomonas* or *Streptococcus* became extinct. This result is an example of the effect that the strength of a single microbial interaction can have on the overall diversity of the community (Ratzke et al., 2020).

Our findings support two scenarios linked to the immigration/elimination model for the lower airways. This model postulates that constant microaspiration and inhaling of microbes is balanced out by mucociliary clearance and local immunity, to maintain a low microbial biomass in the lower airways (Dickson and Huffnagle, 2015). However, when this immigration/elimination balance is disrupted, two scenarios become plausible: (i) otherwise transient immigrants accumulate and consequently lead to an increase in diversity, and (ii) if changes of the local environment favors certain opportunists, they might overtake the community thus causing extinction of other members and a drop in diversity (Dickson and Huffnagle, 2015; Dickson et al., 2015). The first scenario is supported by our finding that Faith's PD increased with the random accumulation of transient genera in the sputum of TB patients. On the other hand, the second scenario would be supported by situations where *Streptococcus* or *Neisseria* became dominant and covered more than 50% of the bacterial content. If there is an optimal window for the *Streptococcus*-to-*Selenomonas*/*Fusobacterium* balance, deviations from it would indicate dysbiotic (unbalanced) states; an argument that should be explored in healthy cohorts to identify such optimal window.

It was striking to find that only among underweight TB patients (BMI ≤ 18.5), abnormal CXRs but neither high mycobacterial load in sputum nor severe clinical findings (TB score ≥6) were associated with changes of the *Streptococcus*-to-*Selenomonas* balance, and therefore with overall compositional variation and Faith's PD. This finding is interesting but not unexpected, considering that abnormal CXRs represent pulmonary damage which is a dramatic change of the local lung environment. It is even more interesting to find that the *Streptococcus*-to-*Selenomonas/Fusobacterium* balance shifted toward increased levels of *Selenomonas* and *Fusobacterium* in patients with lung parenchymal infiltrates when no cavities were observed in CXRs. Lung parenchymal infiltrates/consolidations in adult patients with pulmonary TB have been shown to be severely hypoxic, which exacerbates tissue destruction (Belton et al., 2016). This anaerobic environment plus the availability of nutrients as a result of concomitant tissue destruction are potentially the factors for increased levels of *Selenomonas* and *Fusobacterium*, fastidious gram-negative anaerobes, in parenchymal infiltrates.

At first sight, our findings might disagree with a recent study of *MTB*-infected cynomolgus macaques, where there was no association of compositional changes in the airways microbiome with lung inflammation or involvement (Cadena et al., 2018). However, in that study, subjects were not stratified by BMI. Among participants with normal BMI, we also did not observe compositional changes associated with lung damage. However, in underweight patients, increased levels of *Selenomonas* and *Fusobacterium* were associated with parenchymal infiltrates. A potential explanation for the interaction with BMI, might be provided by the gut-lung axis cross-talk in TB. Based on previous studies, increased levels of certain anaerobes in the gut of underweight TB patients can result in increased levels of short-chain fatty acids (SCFAs) (Maji et al., 2018), which are by-products of anaerobic bacterial metabolism. SCFAs are immuno-modulators that can reach the blood stream and act as anti-inflammatory signaling molecules (Tilg and Moschen, 2015). Furthermore, SCFAs in the lungs can suppress the production of pro-inflammatory cytokines and increase the risk of developing TB in HIV patients with latent TB infection (Segal et al., 2017). Thus, it is plausible that an unbalanced gut microbiome in underweight TB patients might set the immunological tone that favors proliferation of not only *MTB* but also of certain anaerobes in hypoxic lungs. This hypothesis is supported by a previous study reporting an association of impaired cytokine response with low BMI in patients with latent TB (Anuradha et al., 2016).

In our inferred network, *Selenomonas* positively interacts with *Fusobacterium* and with seven other anaerobes (*Campylobacter, Treponema, Schwartzia, Leptotrichia, Magasphera*, unclassified *Veillonellaceae*, and *Bulleidia*), thus forming an anaerobic consortium where *Selenomonas* is a central hub (i.e., high degree node). This finding is in line with previous network inferences reporting *Selenomonas* as a central hub in microbial communities from the oral cavity (i.e., dental plaque) (Faust et al., 2012) and the upper airways (Einarsson et al., 2019). Also, similar anaerobic consortia have been found in colorectal carcinomas (Warren et al., 2013). Even more relevant, *Selenomonas* together with *Veillonella* have been proposed as salivary biomarkers of lung cancer (Yan et al., 2015). Furthermore, in a seminal study, Kolenbrander and colleagues (Kolenbrander et al., 1989) demonstrated that *Selenomonas* organisms (*S. sputigena, S. flueggei, S. infelix*, and *S. noxia*) co-aggregated with *Fusobacterium nucleatum* through cell-to-cell interactions; except for *S.infelix*, we found these species in the sputum of TB patients. Recently, it has been shown that *S. sputigena* has a heavily glycosylated flagella (Rath et al., 2018) which likely contributes to the capability of *Selenomonas spp*. to form multispecies aggregates (biofilms) with members of other genera in dental plaque. Putting together our findings with those of previous studies, there is compelling evidence supporting a key role for *Selenomonas* in shaping the structure of microbial communities in the sputum of TB patients. The implication being that *Selenomonas spp*. potentially migrate to the lower airways as a multispecies co-aggregate of anaerobes which together might tone-down immune responses, probably through production of SCFAs (Mirković et al., 2015; Segal et al., 2017).

Another important finding of this study was that the sputum microbial assemblages of TB patients with HIV were more likely to contain herpesviruses (Epstein-Barr virus) and anelloviruses (Torque Teno virus); in some cases these viruses even dominated the microbial assemblage. This group of viruses are common in human populations, acquired early in life, and are usually non-infectious during a lifetime (Reid et al., 2016). However, these viruses can expand in immuno-compromised individuals, such HIV patients (Miller et al., 2006; Thom and Petrik, 2007; Monaco et al., 2016) and those receiving immunosupressives (Abbas et al., 2017). It is unclear what effect these two types of viruses have on HIV infection particularly in the context of HIV-TB co-infection. Therefore, our finding drives attention to a so far neglected aspect of HIV-TB coinfection, which is the viral component of the airway microbiome.

### 4.1. Limitations

Our findings should be considered in the light of some limitations. First, a large proportion (94%) of TB patients in the cohort reported the previous intake of non-TB medications; patients mostly took penicillin-derivatives (78%). Similar to the previously reported lack of effect of TB treatment on sputum microbial composition (Sala et al., 2020), the distribution of non-TB medications neither differed among the levels of TB-disease manifestations nor were they associated with compositional variation of the sputum microbial communities. Therefore, we are confident that the intake of non-TB medications did not bias the observed associations. Nonetheless, given the well-known impact of medications on human microbiomes, and the serious burden self-medication or miss-treatment poses in low and middle-income countries, we think previous medication-intake should be considered in study designs; excluding participants that received previous medications might neglect the role of medication-intake as a potential mediator or modifying factor in the microbiome-disease interplay in particular study settings.

Second, we followed sputum collection practices to ensure that sputum specimens were produced and collected consistently across participants. These practices included the use of instructional videos, collection of early morning sputum, and qualitative assessment of the specimen's color and viscosity at the time of collection by experienced laboratory technicians. Although these practices were proven to reduce the collection of salivary-like specimens and improve the quality of the sputum for detection of *MTB* (Mhalu et al., 2015), there are additional quantitative measurements that can improve the discrimination of sputum from salivary-like specimens. For instance, the number of squamous epithelium cells or leukocytes, or their ratio, quantified by smear microscopy (Wong et al., 1982). We encourage future studies to incorporate such quantitative measurements to improve the assessment of sputum quality for microbiome studies.

Third, our study observed no correlation between Mycobacterial reads abundance and AFB smear results; likely as a result of our DNA extraction methodology which did not specifically targeted for *MTB* DNA or for removal of host's DNA. We made this decision because both *MTB* DNA enrichment and human DNA depletion would result in distorted compositional profiles of the microbial communities that we are interested in characterizing. For instance, *MTB* enrichment would inevitably affect the estimation of the MTB abundance relative to other microorganisms. On the other hand, due to enzymatic/chemical/mechanical steps to differentially filter/lyse host cells and degrade exposed DNA, human DNA depletion steps can cause an overall loss of microbial DNA or bias toward microorganisms that are less susceptible to such steps. Nonetheless, we must acknowledge that mycobacterial relative abundances estimated from both 16S-AS and WMS-S reads might be an under-representation or miss-representation of the actual *MTB* load in the sputum.

Fourth, even though sputum originates in the lower airways, its passage through the upper airways during expectoration inevitably results in a sample mixed with microbes from the upper respiratory tract and the oral cavity. Therefore, future studies with respiratory samples better rendering the lung lesion environment must verify the reported associations with abnormal CXRs (lung parenchymal infiltrates in particular). Fifth, we report microbial interactions based on the relationships of taxon abundances across a large set of sputum microbial assemblages. However, further experimentation is needed to imply ecological processes such as antagonism or co-existence; for instance, correlations based on absolute abundances measured by quantitative PCRs would confirm the microbial associations reported in this study.

And sixth, our analysis of the WMS-S dataset was limited by the high host-to-microbial DNA ratio. On average 90% of the reads were mapped to a human reference genome and filtered out; remaining reads were not enough for a good coverage of the microbial community and therefore biased against low-abundant members. This bias affects the sensitivity of functional profiling methods to estimate the contribution of microbial species with low-covered genomes to a functional pathway or to a gene family. For this reason we decided to limit our analysis of the WMS-S dataset to perform only a species-level taxonomic profiling that could complement the 16S-AS profiles.

### 4.2. Conclusions

In summary, this study sheds light on the relationship between pulmonary TB and the host's airway microbiome. We have identified specific microbial interactions responsible for structuring the microbial communities in the sputum of TB patients. More importantly, our results suggest underweight nutritional status (BMI ≤ 18.5) as a determinant factor for sputum microbial associations with hypoxic lesions in pulmonary TB. Thus, our hypothesis that variations in TB disease manifestations are associated with microbial composition variation in the airways of TB patients, partly holds; the association is restricted to pulmonary damage and determined by underweight status. Finally, but importantly, our study points out a non explored nexus between TB and HIV, the airways virome. These new insights should guide further research to unravel the underlying mechanisms behind the observations presented here.

## Data Availability Statement

The datasets (raw sequence reads) supporting the conclusions of this article are available in the SRA repository, under BioProject ID PRJNA532879 at the given link https://www.ncbi.nlm.nih.gov/bioproject/532879. Jupyter notebooks with the computational and statistical analyses are available in the GitLab repository under the link https://git.scicore.unibas.ch/TBRU/tbdarbiome_cases.git. Notice that the GitLab repository does not contain patient sensitive data; an extended version of the GitLab repository, with anonymized patient data, is archived in Zenodo under the following link http://doi.org/10.5281/zenodo.4281142.

## Ethics Statement

The studies involving human participants were reviewed and approved by the Ifakare Health Institute-Institutional Review Board (IHI-IRB) and the National Health Research and Ethics Committee at the National Institute for Medical Research in Tanzania. The patients/participants provided their written informed consent to participate in this study.

## Author Contributions

MT analyzed and interpreted data. JH collected and managed epidemiological data. MT and SG wrote the manuscript. JH, HH, and MS enrolled participants, collected, and processed samples in Tanzania. FM collected and managed diagnostic data for respiratory and helminth infections. SS processed samples in Switzerland. CB performed whole metagenome shotgun sequencing. LR performed the genotyping of *MTB* clinical isolates. LF, MH, IC, and SD developed the concept. LF, KR, and SG procured the funding. All authors read and approved the final manuscript.

## Conflict of Interest

The authors declare that the research was conducted in the absence of any commercial or financial relationships that could be construed as a potential conflict of interest.

## Publisher's Note

All claims expressed in this article are solely those of the authors and do not necessarily represent those of their affiliated organizations, or those of the publisher, the editors and the reviewers. Any product that may be evaluated in this article, or claim that may be made by its manufacturer, is not guaranteed or endorsed by the publisher.
