# Supplementary material for: The Sputum Microbiome in Pulmonary Tuberculosis and Its Association With Disease Manifestations: A Cross-Sectional Study
Source: Front Microbiol. 2021 Aug 20;12:633396. doi: 10.3389/fmicb.2021.633396 (PMC8417804; doi:10.3389/fmicb.2021.633396)
Supplement: Supplementary file 1 [file Data_Sheet_1.PDF]

Supplementary Material

1 SUPPLEMENTARY TABLES AND FIGURES

1.1 Figures

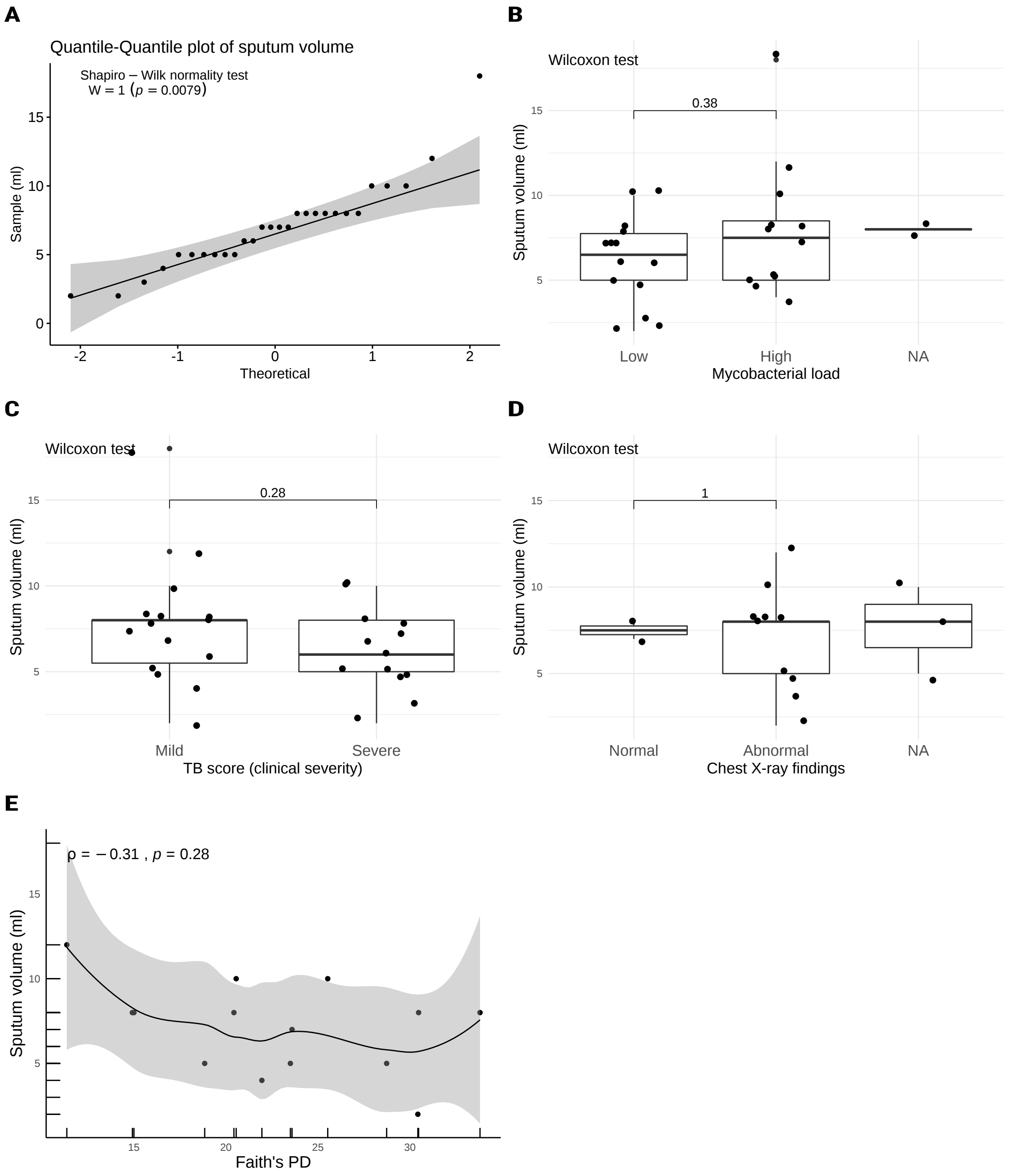

Figure S1. Distribution of sputum volume by categories of TB-disease manifestations. **(A)** Quantile-Quantile plot of sputum volume shows the non-normal distribution. Sputum volume by Mycobacterial load (B), by Clinical severity (C), and by chest X-rays findings (D). Non-significant correlation between sputum volume and Faith's phylogenetic diversity.

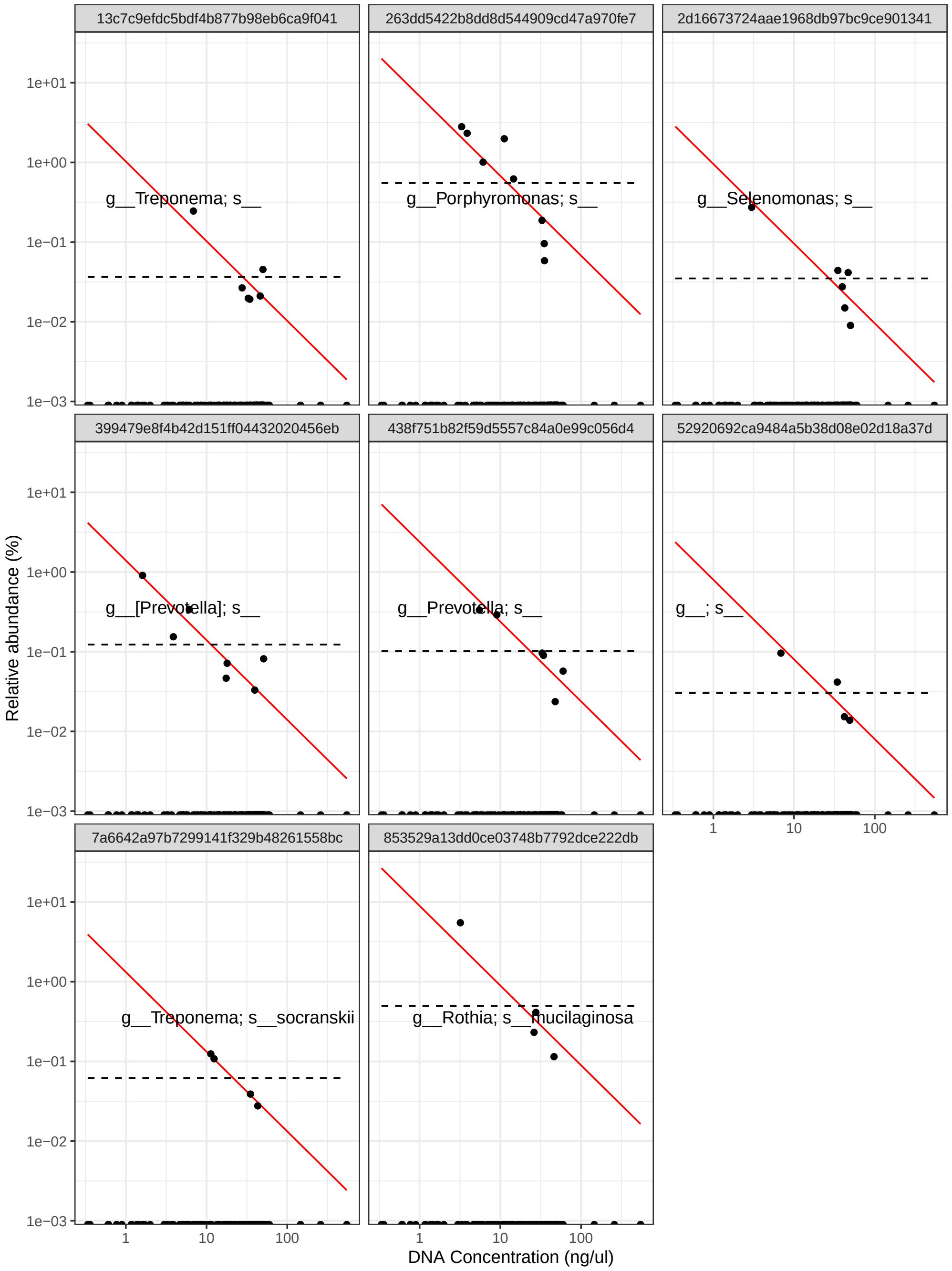

Figure S2. Potential contaminants in the 16S-AS dataset. Using the DNA concentration of sputum samples, ASVs whose frequency varied inversely with total DNA concentration and detected in at least four samples were flagged as contaminants by *R* package *decontam*.

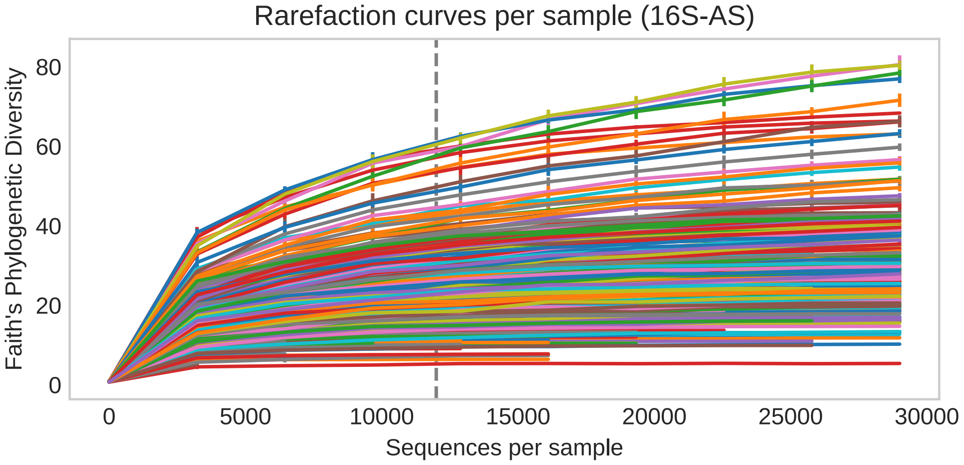

Figure S3. Rarefaction curves of Faith's phylogenetic diversity as a function of the number of sequences per sample in the 16S-AS dataset. The horizontal dashed line corresponds to a threshold of 12,000 sequences per sample

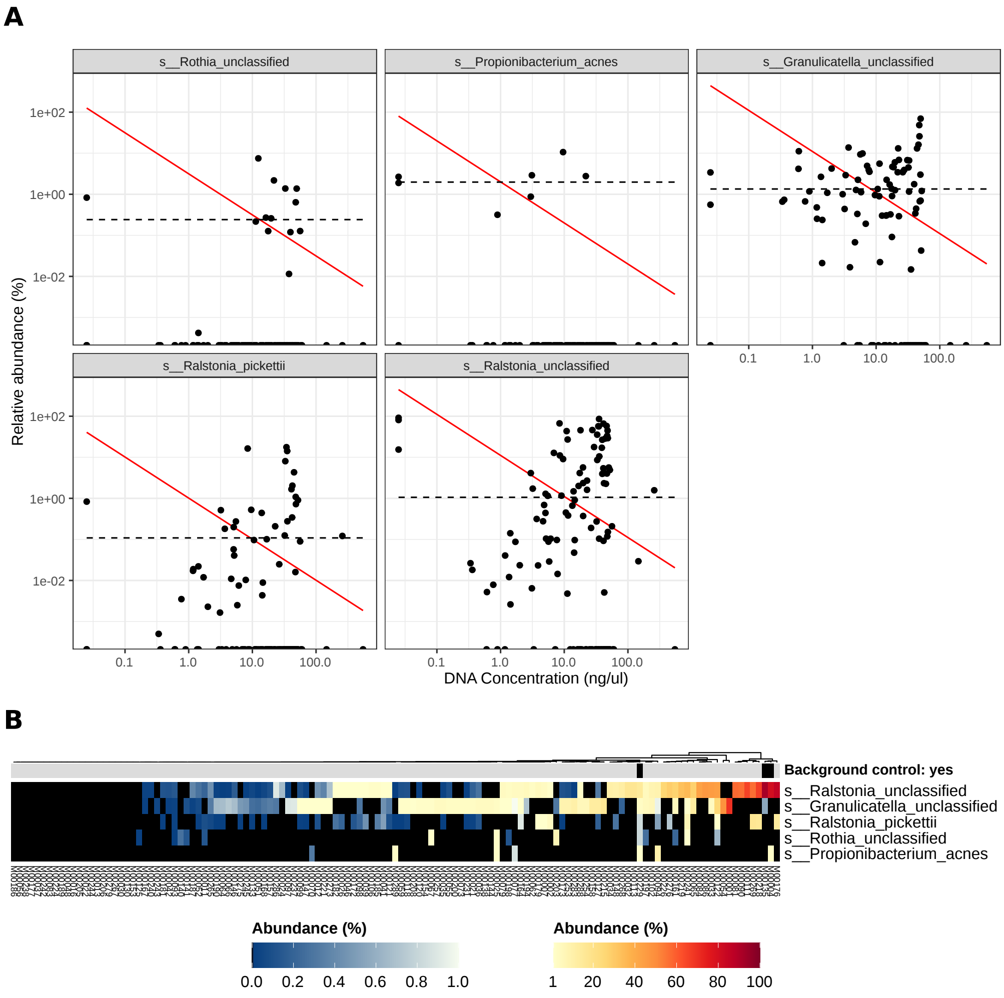

Figure S4. Potential contaminants in the WMS-S dataset.We used the DNA concentration of sputum samples and background controls to identify potential contaminants with *R* package *decontam*. Species whose frequency varied inversely with total DNA concentration (A) or were present in background controls (B) were flagged as contaminants by *decontam*. Samples with more than 50% reads assigned to contaminant species were removed from further analysis.

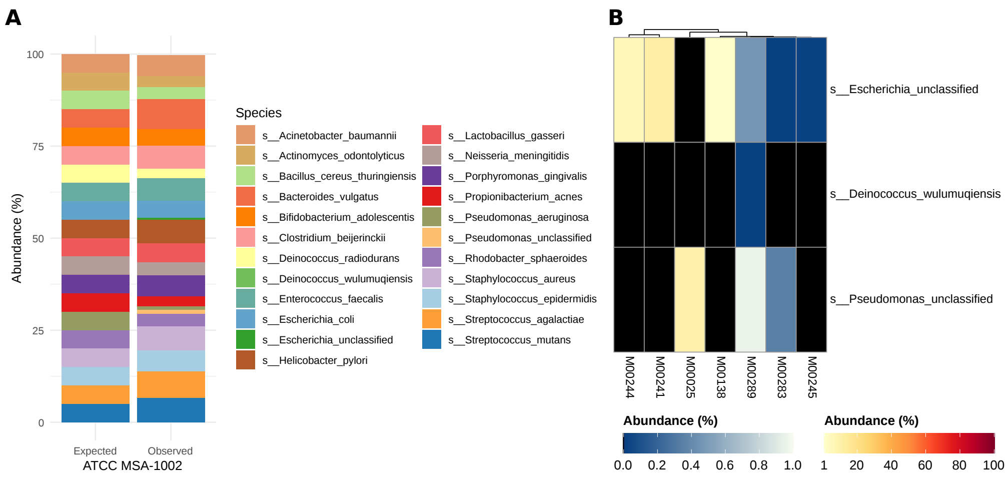

Figure S5. Consistency of WMS-S taxonomic profiles with mock community. (A) observed versus expected taxonomic composition of a mock community screened by WMS-S. (B) Abundance of non-expected species observed in the mock community and in a few sputum samples.

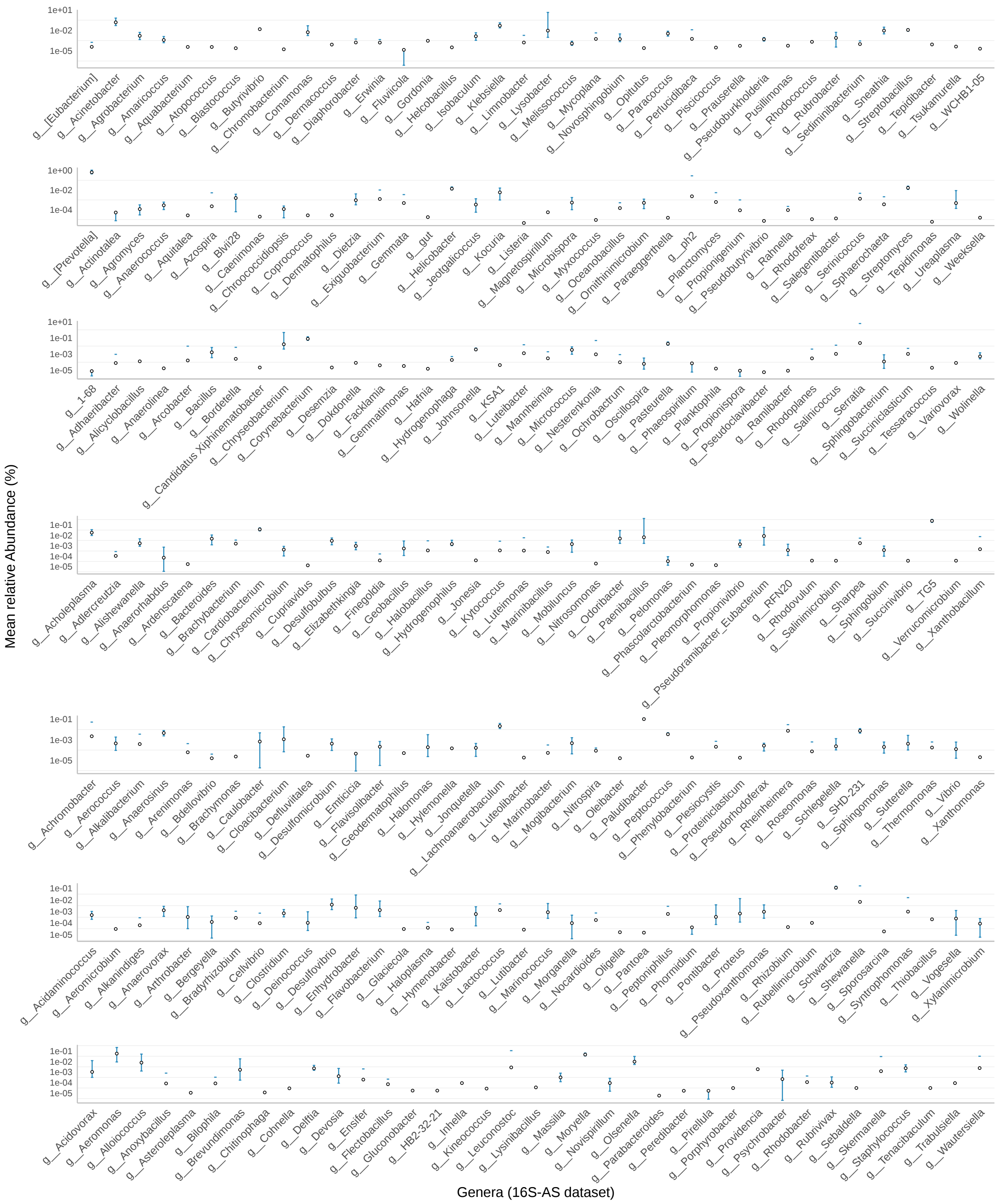

Figure S6. Mean relative abundance (%) of genera not detected through WMS-S. Mean relative abundances, obtained from the 16S-AS dataset, are represented as black circles. Blue lines represent non-parametric studentized bootstrap 95% confidence intervals. The y-axis is log10 transformed, and does not display negative values of confidence intervals.

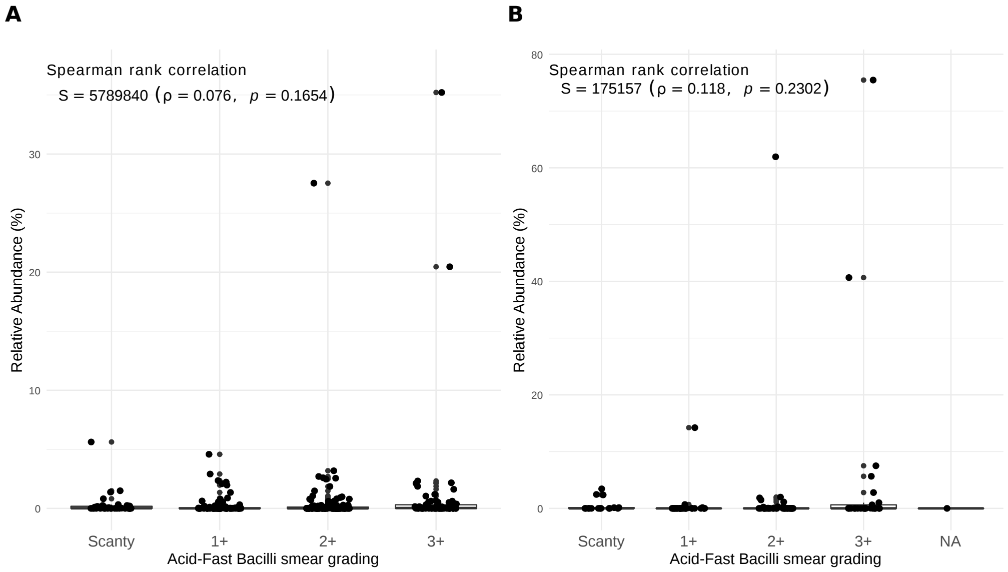

Figure S7. Non-correlation of putative *Mycobacterium tuberculosis* with AFB smear grading. (A) Mycobacterium ASV detected in 16S-AS profiles. (B) *Mycobacterium tuberculosis* complex species identified by MetaPhlAn2 in the WMS-S profiles. Within parenthesis, the correlation coefficient and the corresponding p value.

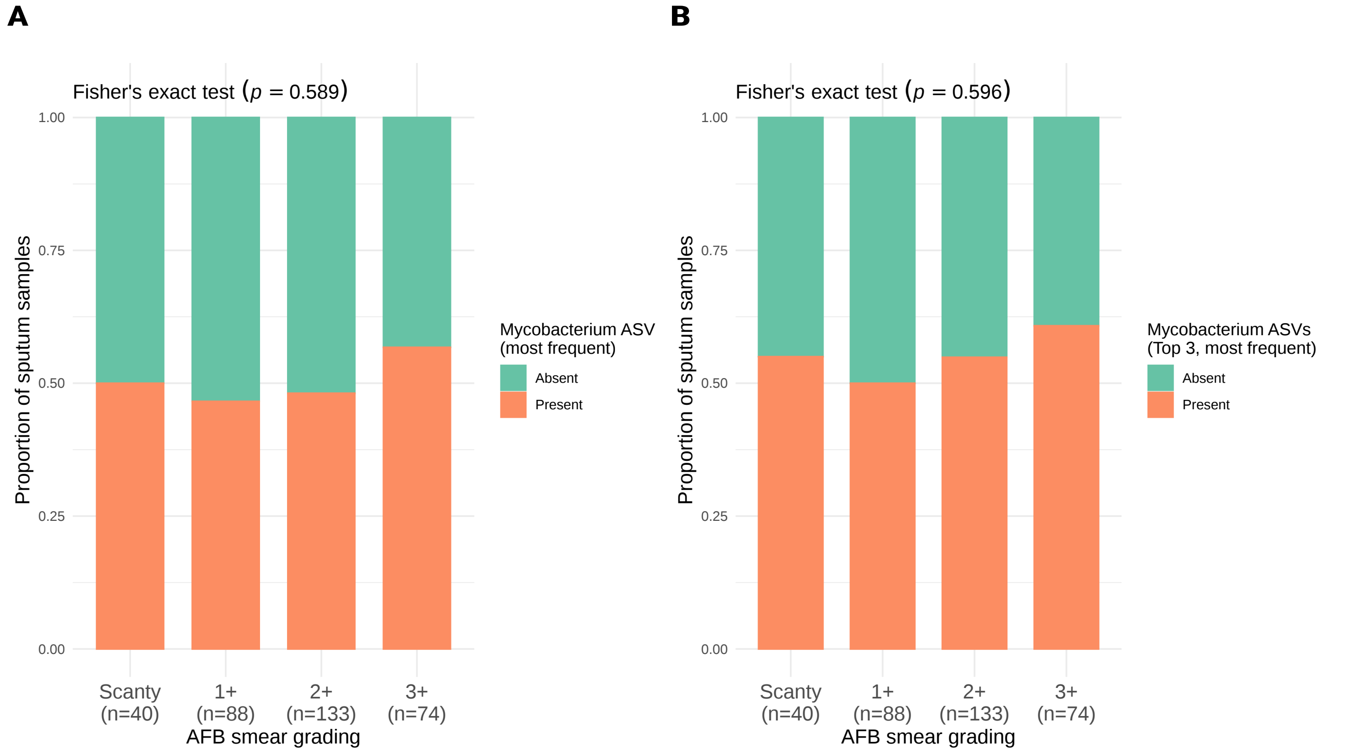

Figure S8. Non-association between AFB smear grading and detection of Mycobacterium Amplicon Sequence Variants(ASVs). **(A)** Detection of the most frequent *Mycobacterium* ASV by AFB smear grading. **(B)** Detection of the top 3 most frequent *Mycobacterium* ASVs by AFB smear grading.

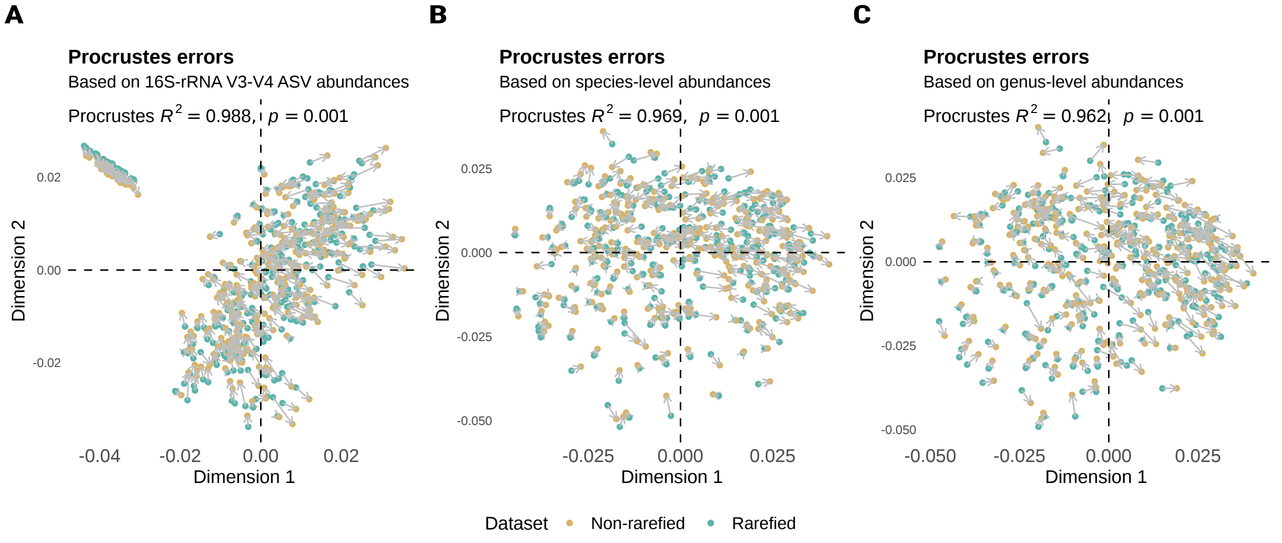

Figure S9. Procrustes rotation analysis of ordinations based on rarefied and non-rarefied 16S-AS taxonomic profiles. Procrustes rotations of ordinations based on centred log-ratio transformed abundances of Amplicon Sequence Variants (ASVs) (**A**), species (**B**), and genera (**C**). Analysis available at `https://git.scicore.unibas.ch/TBRU/tbdarbiome_cases/-/blob/master/notebooks/09_MOT_rarefied-vs-nonrarefied-coda.ipynb`

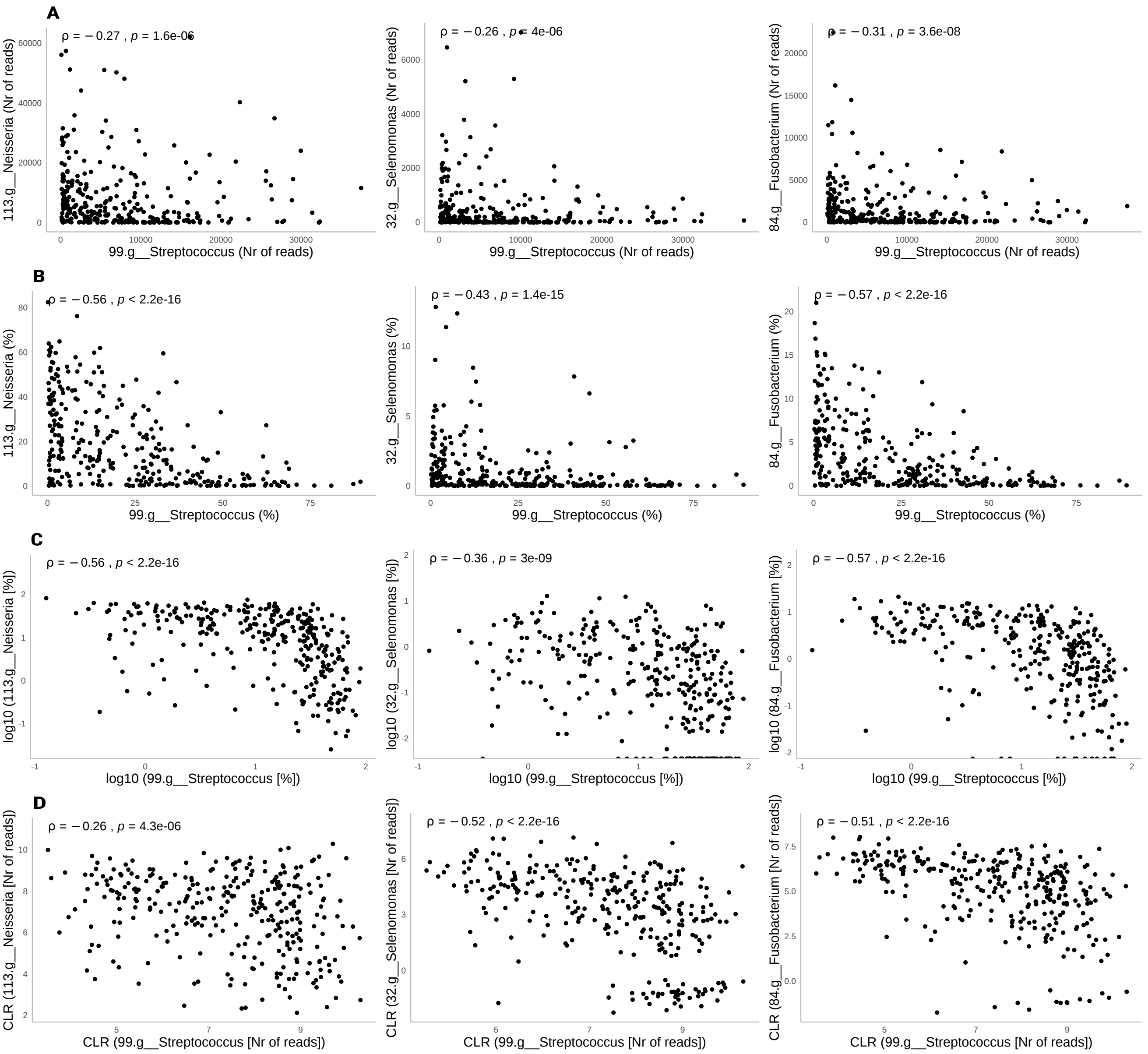

Figure S10. Negative correlations between *Streptococcus* and Neisseria, and anaerobes Selenomonas and Fusobacterium. (**A**) shows the relative abundance (%) of Streptococcus against Neisseria, Selenomonas, and Fosobacterium. (**B**) shows the same plots as in A but in logarithmic scale.

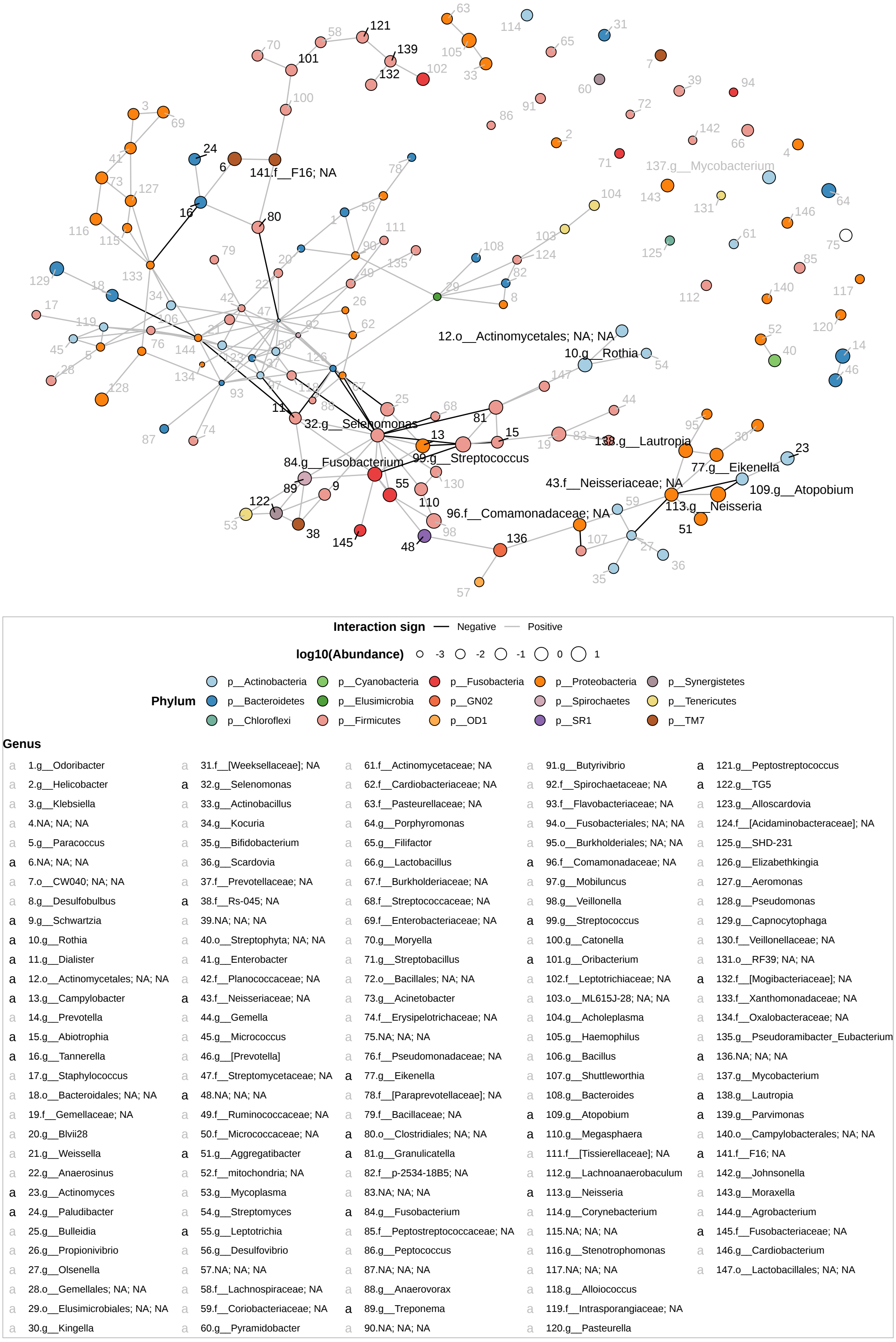

Figure S11. Genus-level interaction network inferred with SPIEC-EASI. Nodes represent genera, colored by the corresponding phylum. Node sizes correspond to the relative abundance (%) averaged across sputum microbial assemblages and in logarithmic scale. To avoid overcrowding the figure with text labels, we used numeric labels for most nodes. We specified the corresponding taxonomic identity in the legend 'Genus' which includes the number and the genus name, or the family name, or the order name if any of the previous ones were not assigned. To infer the network, we included genera detected in at least 5% of the sputum samples profiled by 16S-rRNA-gene amplicon sequencing.

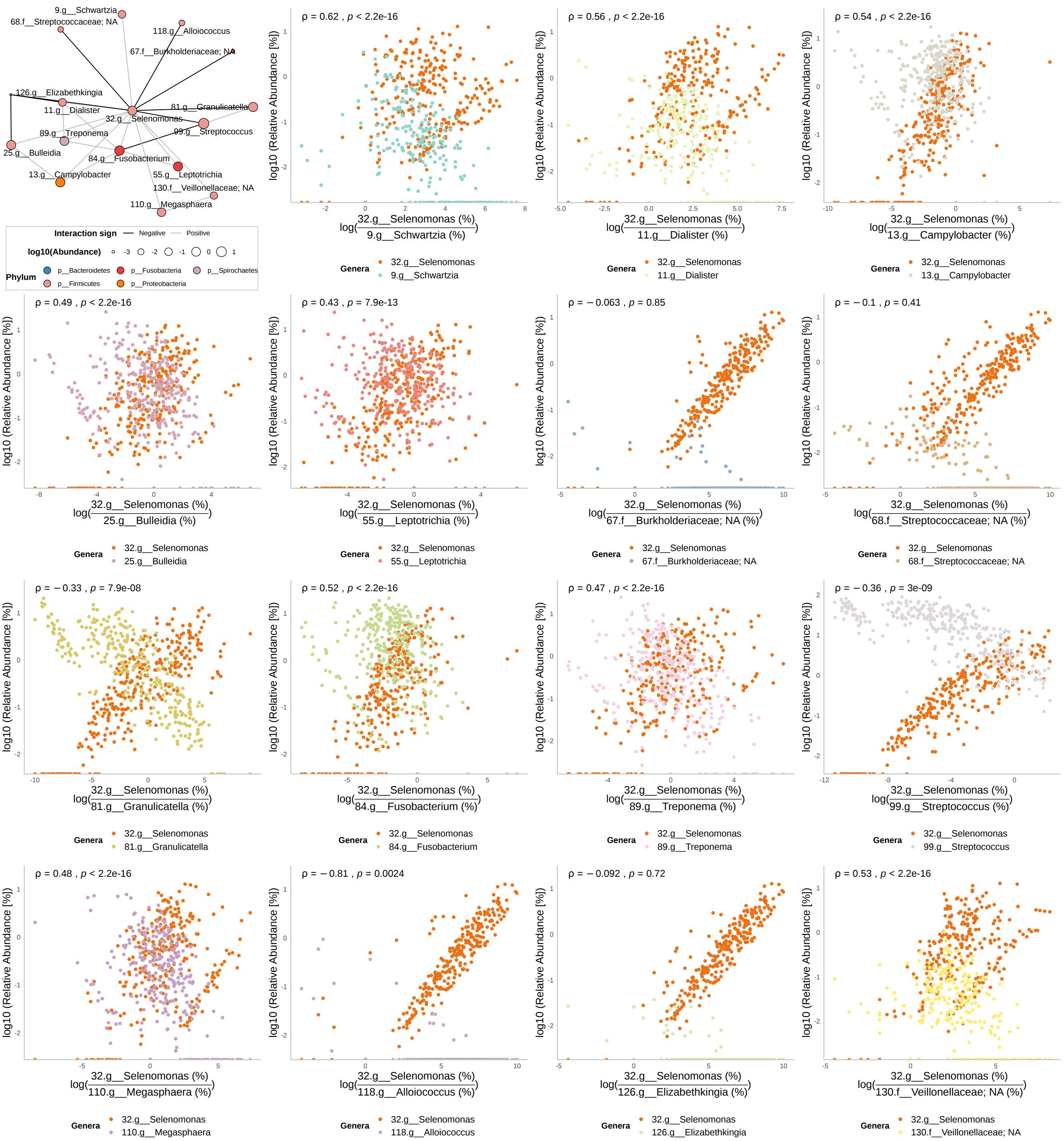

Figure S12. Neighborhood of *Selenomonas* in the genus level interaction network. The relationship between *Selenomonas* and other genera is shown by plotting the abundance log-ratio of *Selenomonas* and the genus interacting with it (x-axis), and the corresponding relative abundance of each genus, in log scale (y-axis). We computed the Pearson correlation coefficient (R) to assess correlations between the log abundances of every pair of interactions.

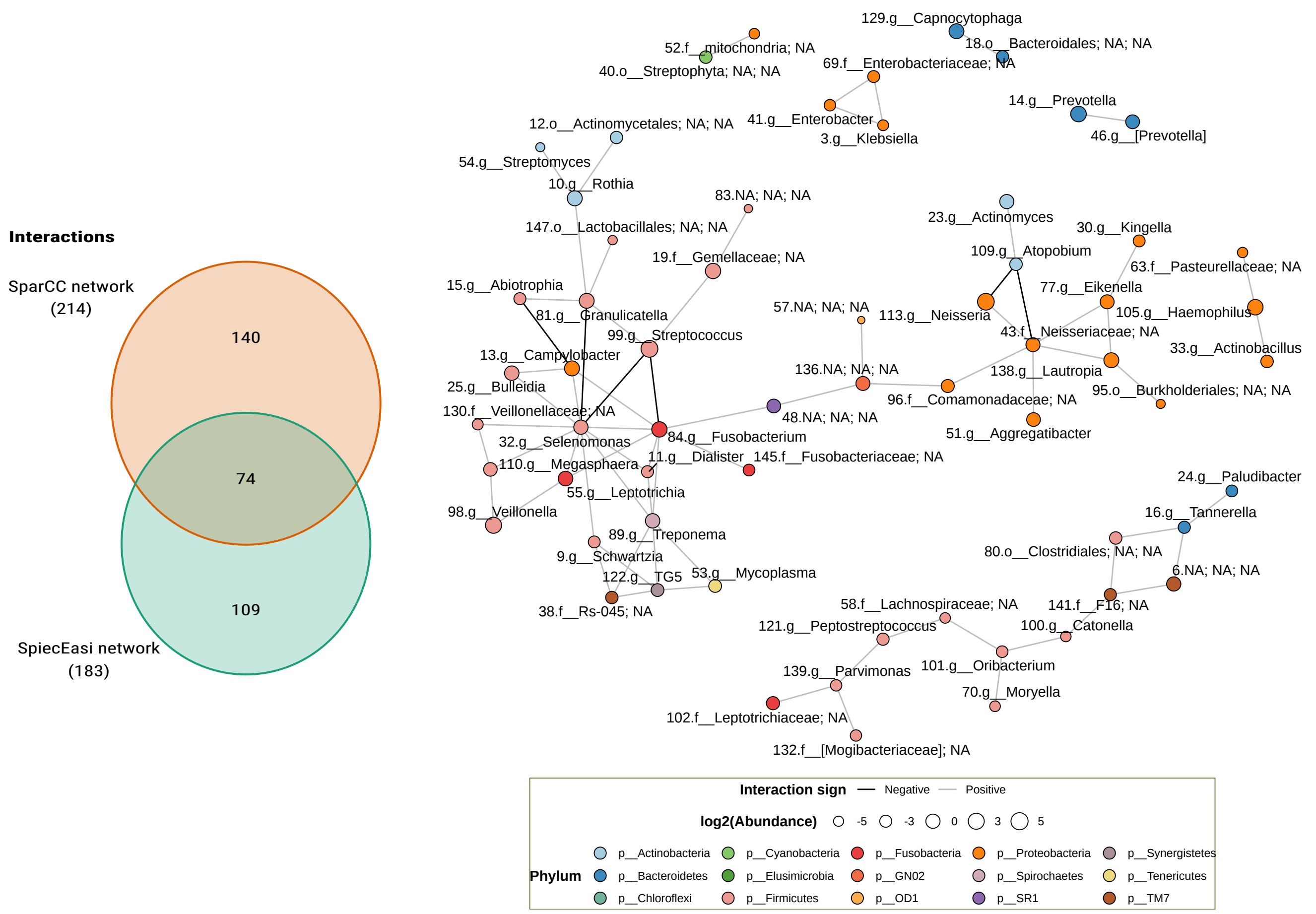

Figure S13. Genus-level microbial association networks inferred by SparCC and SpiecEasi using the 16S-AS dataset. Left, Venn diagram of interactions in the SparCC and SpiecEasi networks. Right, network with interactions inferred by both SparCC and SpiecEasi.

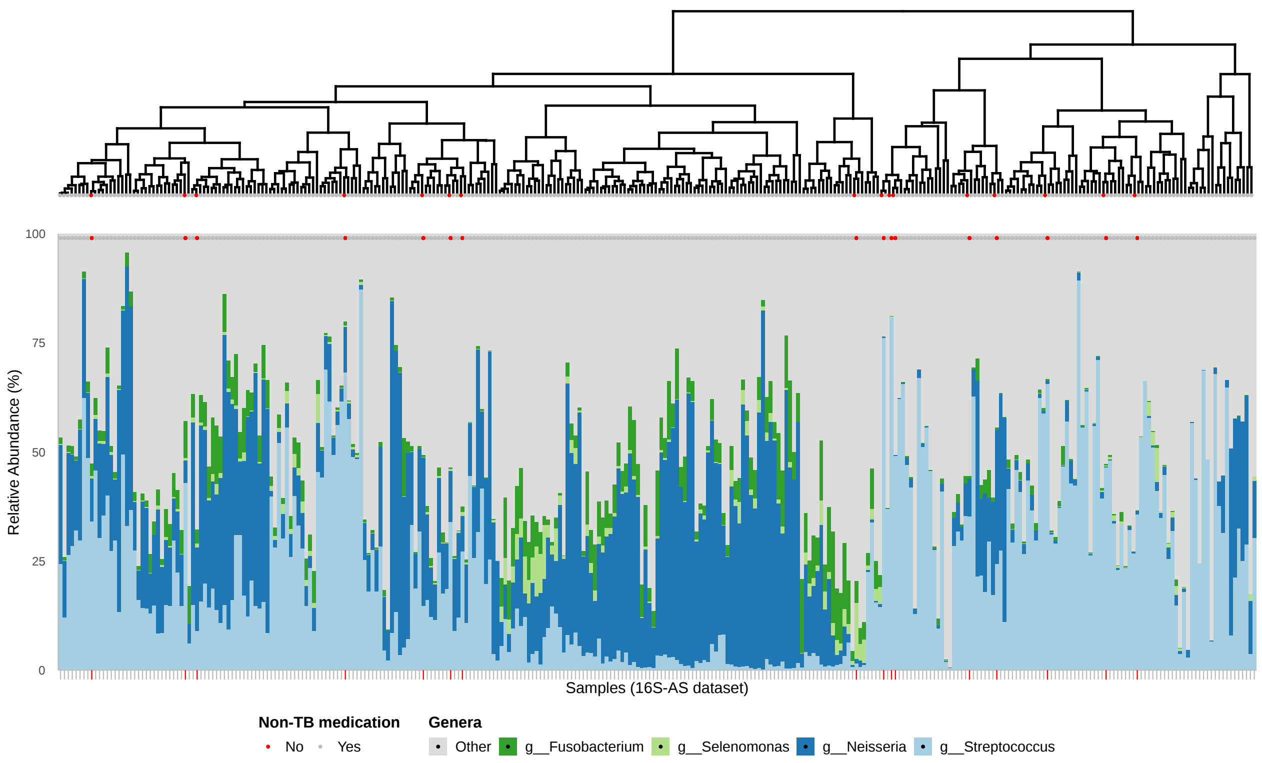

Figure S14. Bar plot of the relative abundance of genera *Streptococcus*, *Neisseria*, *Selenomonas*, and *Fusobacterium*. Samples were sorted according to hierarchical clustering based on Aitchison distances.

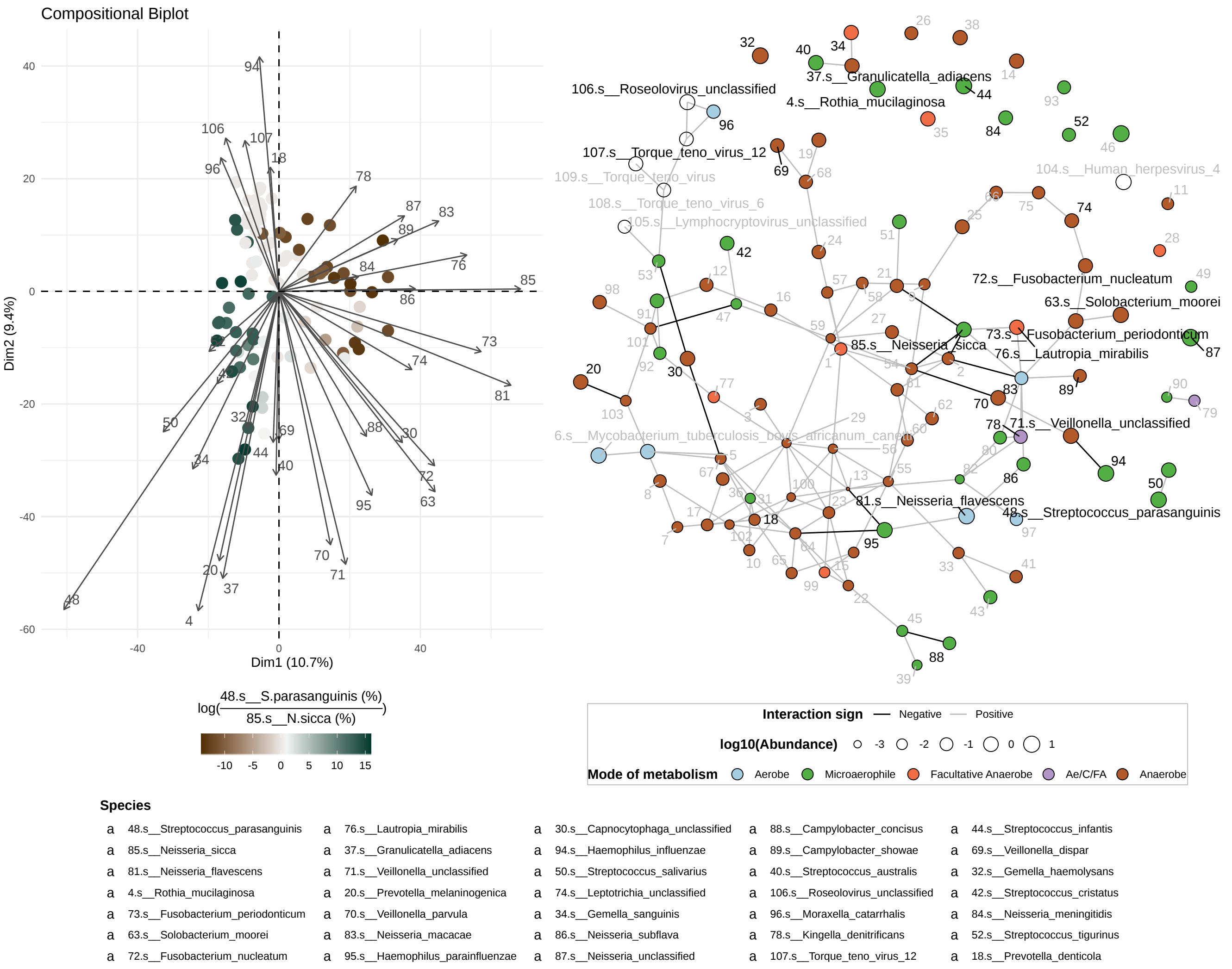

Figure S15. Species-level community interactions in the sputum of TB patients. Left, relative variation biplot of species-level community compositions (centred log-ratio transformed abundances). The biplot shows the relative variation of the log-ratios for the top 35 taxa (arrows, taxa best represented by the axes of the biplot), and sputum samples (dots); the length of the imaginary line connecting the tips of two arrows, representing two taxa, is proportional to the variation of the log-ratio of the corresponding abundances; sputum samples are colored by the *Streptococcus parasanguinis*-to-*Neisseria sicca* abundance log-ratio. Right, interaction network inferred with the SPIEC-EASI method. Nodes represent species, colored by the corresponding mode of metabolism, and of sizes corresponding to the relative abundance (%) averaged across sputum microbial assemblages and in logarithmic scale. To avoid overcrowding the figures with text labels, we used numeric labels for arrows and nodes; for the interaction network, we highlighted in bold the taxa displayed in the compositional biplot. We specified the corresponding taxonomic identity in the legend 'Species' which includes the number and the species name (see Fig. S16 for the complete set of labels). To create the compositional biplot and to infer the network, we included species detected in at least three sputum samples profiled by WMS sequencing.

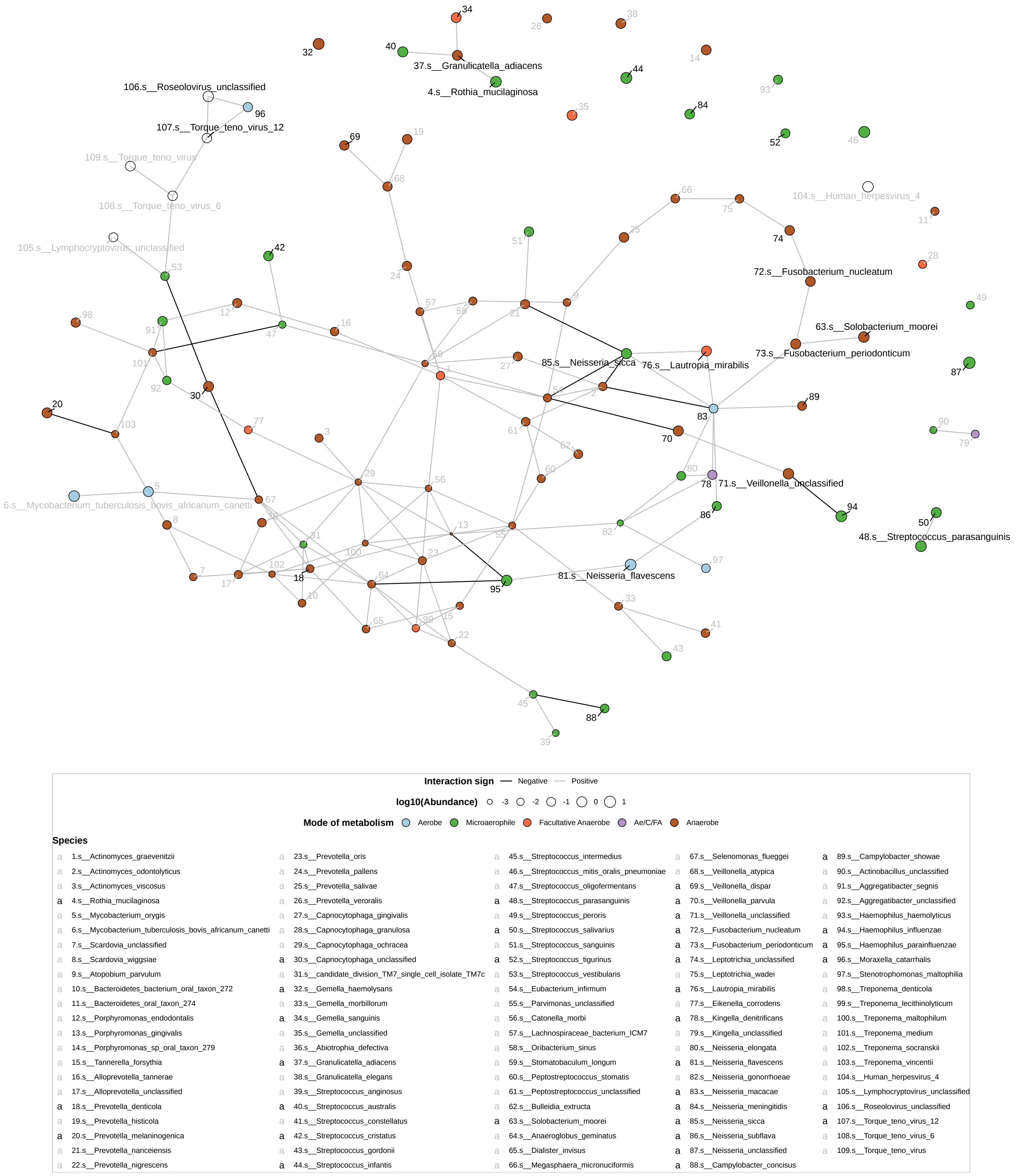

Figure S16. Species-level interaction network inferred with SPIEC-EASI. Nodes represent species, colored by the corresponding mode of metabolism, and of sizes corresponding to the relative abundance (%) averaged across sputum microbial assemblages and in logarithmic scale. To avoid overcrowding the figure with text labels, we used numeric labels for most nodes. We specified the corresponding taxonomic identity in the legend 'Species', as assigned by MetaPhlAn2.

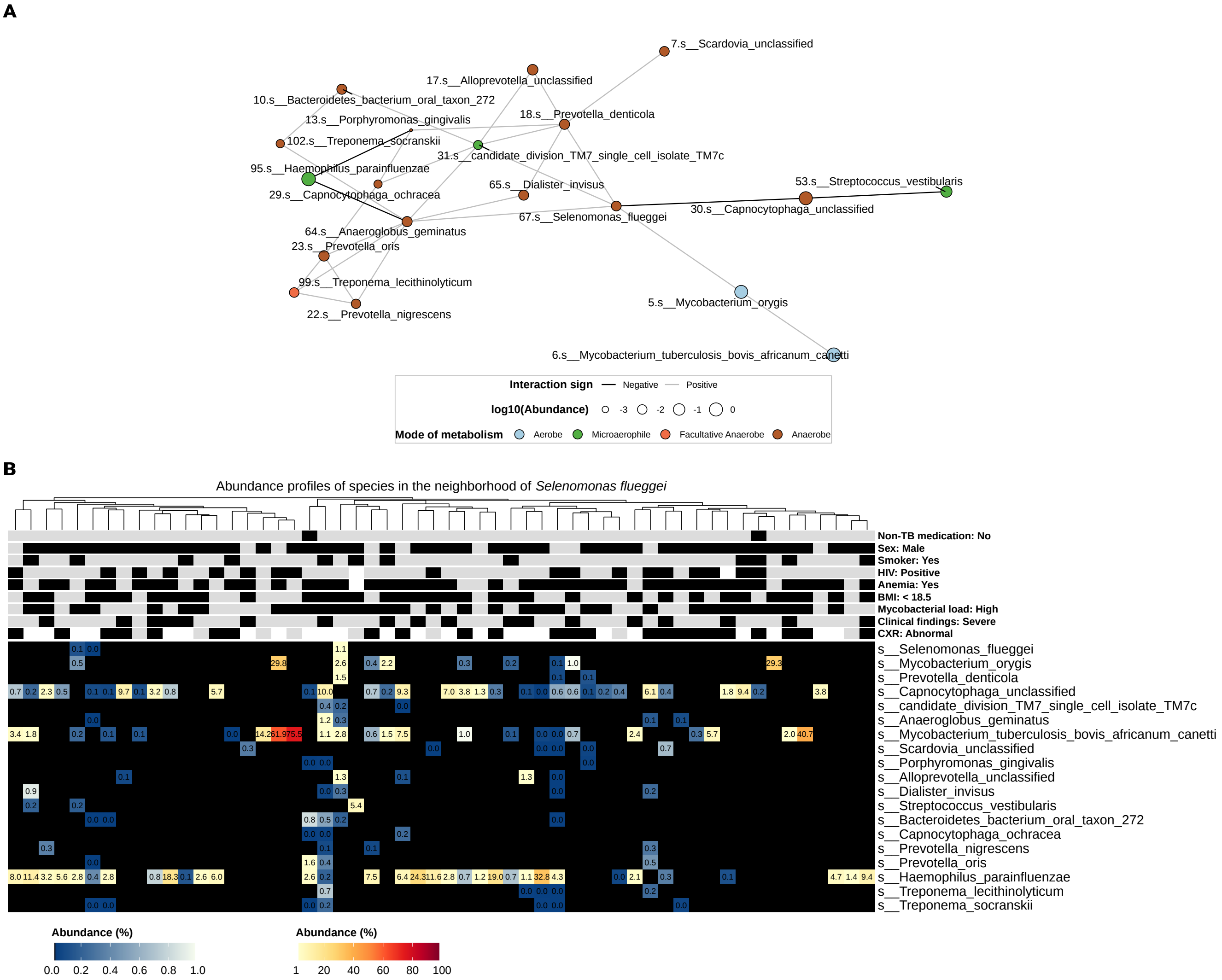

Figure S17. Species-level microbial associations of *Selenomonas spp.* **(A)** Neighborhood of *Selenomonas spp.* in the species-level interaction network inferred by SpiecEasi. **(B)** Heatmap of *Selenomonas spp.* and its interacting neighbors. The network and heatmap were created using the WMS-S dataset.

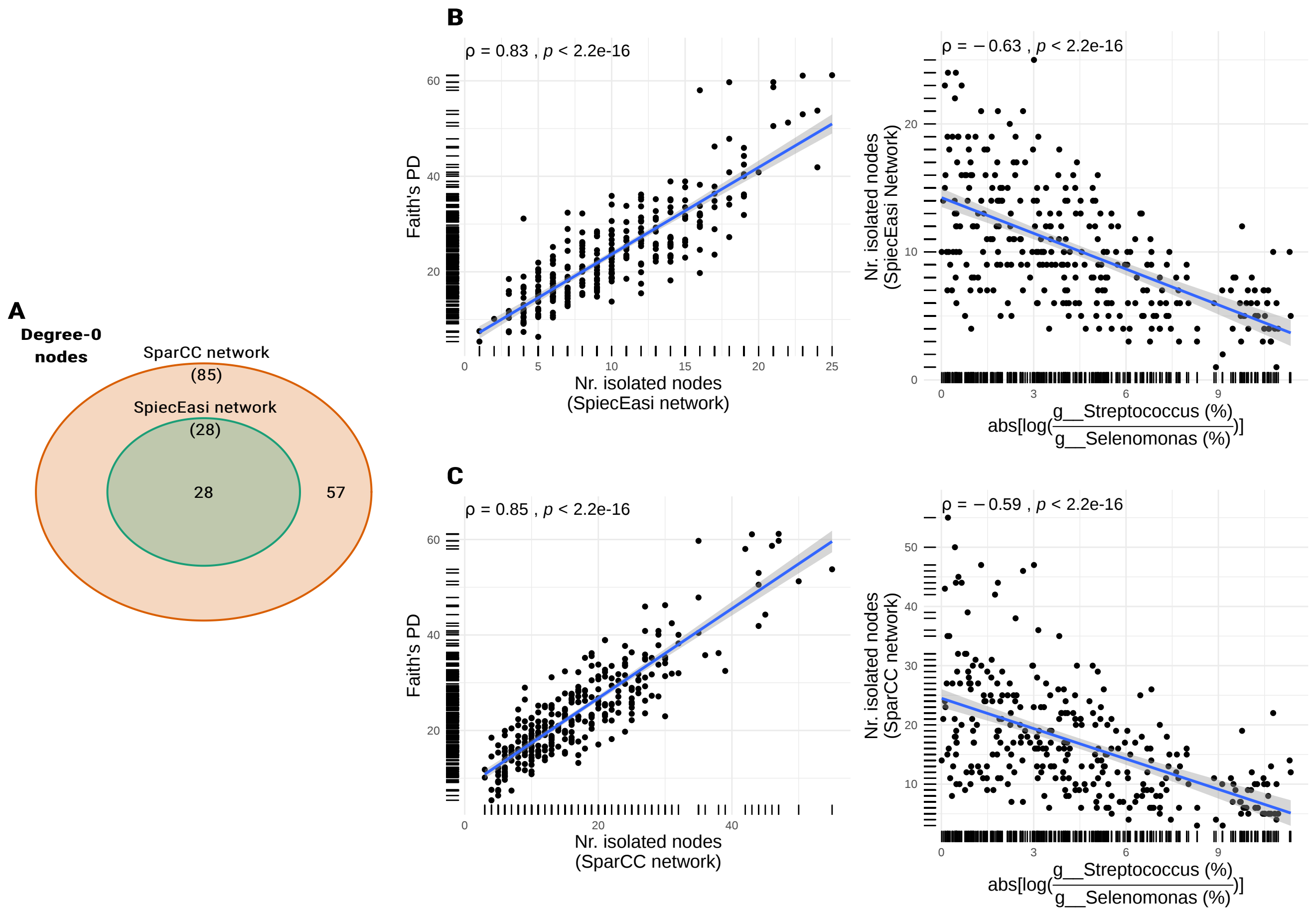

Figure S18. Associations with accumulation of isolated nodes in genus-level microbial association networks. **(A)** Venn diagram of isolated nodes in genus-level interaction networks inferred by SparCC and SpiecEasi. Accumulation of isolated nodes in sputum samples was correlated with Faith's PD and with the strength of the Streptococcus-Selenomonas interaction; for both the SpiecEasi **(B)** and the SparCC **(C)** networks.

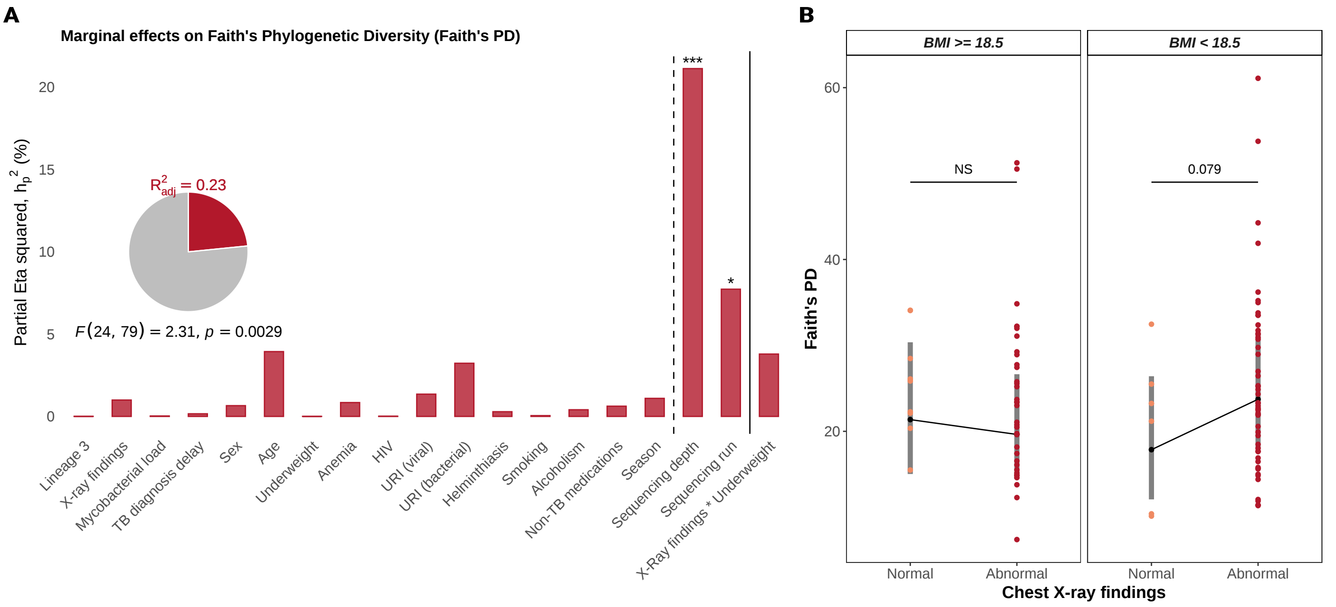

Figure S19. Associations with Faith's phylogenetic diversity (PD) when considering the *Mycobacterium tuberculosis* complex lineage. (**A**) Marginal effect sizes of variables included in multi-way ANCOVA model testing associations with logarithm-transformed Faith's PD; effect sizes correspond to the partial Eta squared statistic (h_p^2); the adjusted R-squared statistic, the model's F statistic, and the significance of the model and of the variables are shown: * ($p \leq 0.05$), ** ($p \leq 0.01$), *** ($p \leq 0.001$), and **** ($p \leq 0.0001$). (**B**) Plot showing the estimated marginal means of Faith's PD by Chest x-ray findings and underweight status, based on the ANCOVA model to the left; grey bars correspond to 95% confidence intervals of the estimated means.

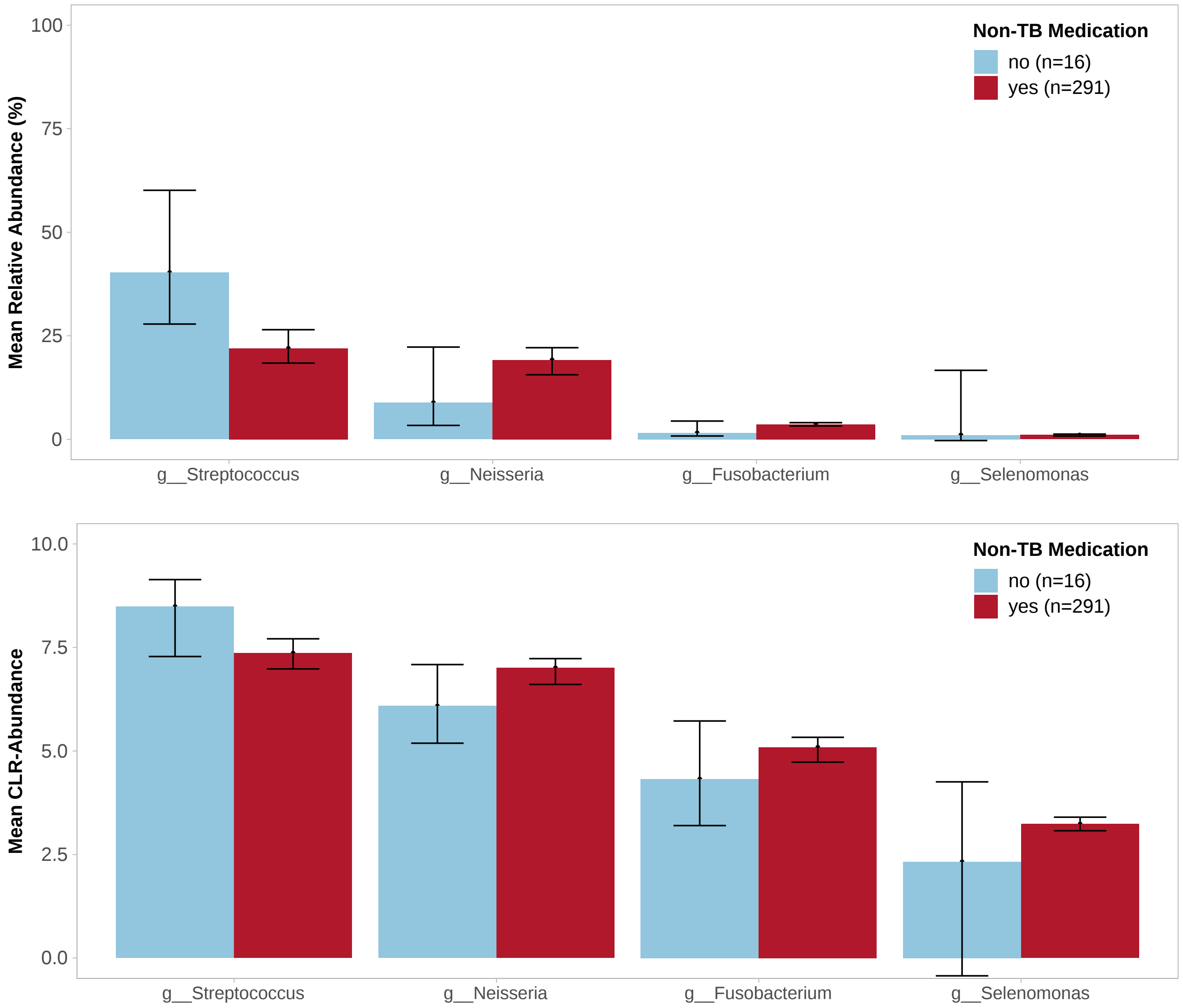

Figure S20. Abundance of genera *Streptococcus*, *Neisseria*, *Fusobacterium*, and *Selenomonas* by exposure to non-TB medications. **(Top)** shows how differences in mean relative abundances (%) were only significant for genus *Streptococcus*. However, **Bottom** shows that after CLR-transformation of relative abundances, to avoid spurious differences due to compositional bias, differences were not significant. Differences were considered significant when 95% studentized bootstrapped confidence intervals did not overlap.

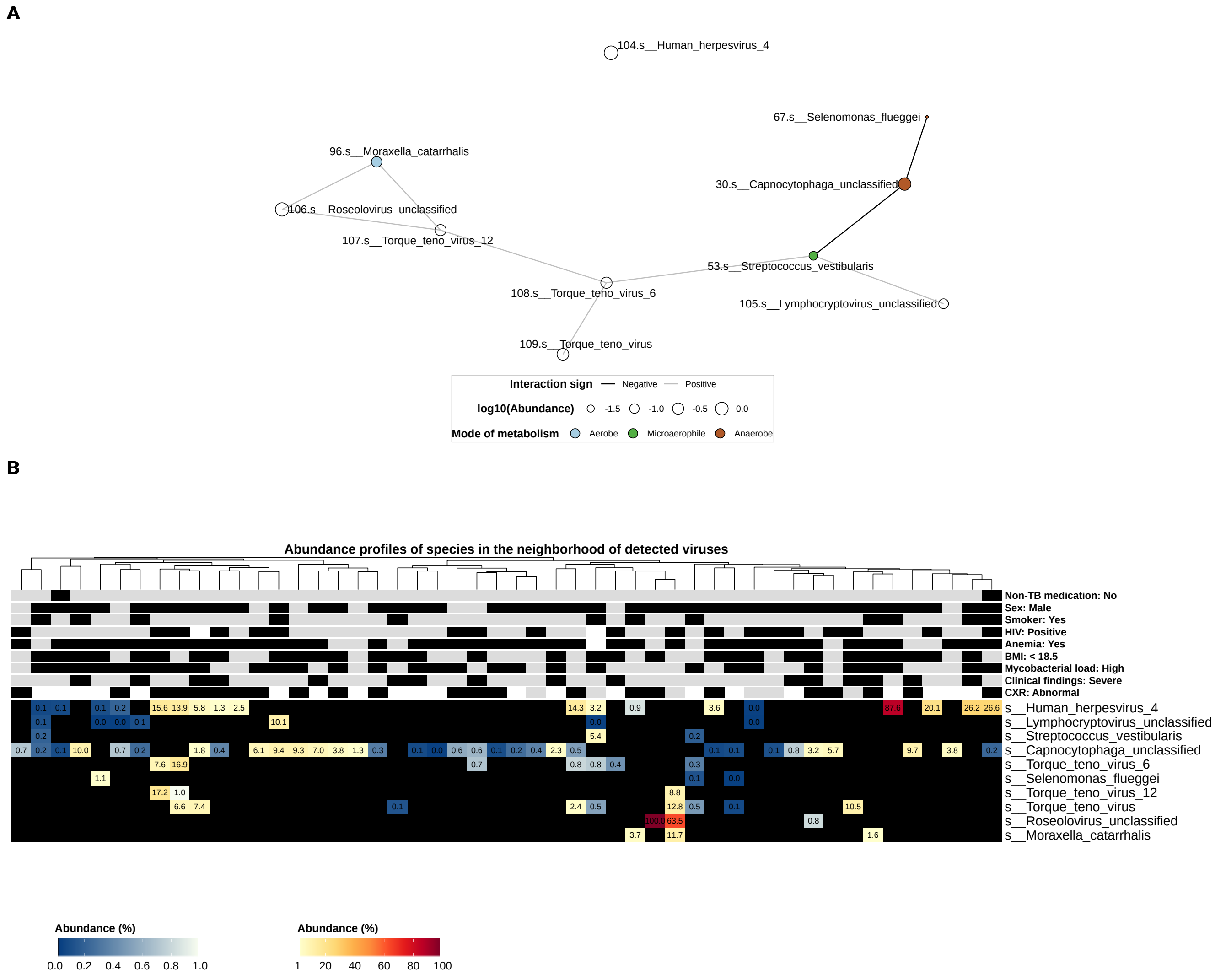

Figure S21. Associations between viruses and bacterial species. (**A**) SpiecEasi inference of associations between viruses and bacterial species, using the WMS-S dataset. **(B)** Heatmap of relative abundances for viruses and its interacting neighbors.

1.2 Tables

Table S1. Characteristics of TB patients by presence of infiltrates or cavities in chest x-rays

Characteristics	**Total** (N=334)	**Infiltrates**[e] Absent (N=91)	Present (N=156)	p.value[a]	**Cavities**[e] Absent (N=159)	Present (N=88)	p.value[a]
**Demographics**							
**Sex**				**0.002**			0.400
Male	240 (71.86%)	51 (56.04%)	119 (76.28%)		106 (66.67%)	64 (72.73%)	
Female	94 (28.14%)	40 (43.96%)	37 (23.72%)		53 (33.33%)	24 (27.27%)	
**Age** (years)				0.097			0.075
Median (IQR)	33.00 (14.00)	34.00 (13.50)	32.00 (15.00)		33.00 (16.00)	31.00 (13.25)	
**Physical health parameters**							
**BMI** (kg/m2)				0.031			<**0.001**
Median (IQR)	18.26 (3.85)	19.15 (3.49)	18.00 (3.59)		18.90 (3.97)	17.71 (3.27)	
**Nutritional status**				0.010			0.005
Normal weight	145 (43.41%)	51 (56.04%)	62 (39.74%)		81 (50.94%)	32 (36.36%)	
Underweight	176 (52.69%)	34 (37.36%)	89 (57.05%)		68 (42.77%)	55 (62.50%)	
Obesity	13 (3.89%)	6 (6.59%)	5 (3.21%)		10 (6.29%)	1 (1.14%)	
**Hemoglobin** (g/dL)				0.337			0.992
Median (IQR)	11.25 (2.92)	10.80 (3.25)	11.30 (2.65)		11.15 (3.28)	11.05 (2.60)	
Missing	18	8	5		9	4	
**Anemia status**				>0.999			0.173
No	81 (25.63%)	21 (25.30%)	37 (24.50%)		42 (28.00%)	16 (19.05%)	
Yes	235 (74.37%)	62 (74.70%)	114 (75.50%)		108 (72.00%)	68 (80.95%)	
Missing	18	8	5		9	4	
**Respiration rate** (breaths/min)				0.772			0.387
Median (IQR)	17.00 (5.00)	17.00 (4.50)	18.00 (4.00)		17.00 (5.00)	18.00 (4.00)	
**Smoker**				0.245			0.325
no	280 (83.83%)	83 (91.21%)	133 (85.26%)		142 (89.31%)	74 (84.09%)	
yes	54 (16.17%)	8 (8.79%)	23 (14.74%)		17 (10.69%)	14 (15.91%)	
Cigarettes per day				0.855†			0.212†
Median (IQR)	7.00 (6.25)	6.00 (3.25)	7.00 (5.00)		6.00 (3.00)	9.50 (6.00)	
**Alcohol abuse**				0.065			0.288
no	269 (80.54%)	80 (87.91%)	121 (77.56%)		133 (83.65%)	68 (77.27%)	
yes	65 (19.46%)	11 (12.09%)	35 (22.44%)		26 (16.35%)	20 (22.73%)	
**Co-Infections**							
HIV				<**0.001**			<**0.001**
negative	244 (76.49%)	46 (54.12%)	131 (86.18%)		103 (67.32%)	74 (88.10%)	
positive	75 (23.51%)	39 (45.88%)	21 (13.82%)		50 (32.68%)	10 (11.90%)	
Missing	15	6	4		6	4	
CD4+ T cell counts (cells/ul)				0.289			>0.999
<200	22 (52.38%)	13 (50.00%)	7 (77.78%)		18 (58.06%)	2 (50.00%)	
>=200	20 (47.62%)	13 (50.00%)	2 (22.22%)		13 (41.94%)	2 (50.00%)	
Previous ART				0.123‡			>0.999‡
No	309 (94.50%)	80 (91.95%)	151 (96.79%)		147 (94.84%)	84 (95.45%)	
Yes	18 (5.50%)	7 (8.05%)	5 (3.21%)		8 (5.16%)	4 (4.55%)	
Missing	7	4	0		4	0	
**Respiratory viruses**[b]				0.473			0.260
no	227 (78.82%)	64 (81.01%)	100 (75.76%)		111 (80.43%)	53 (72.60%)	
yes	61 (21.18%)	15 (18.99%)	32 (24.24%)		27 (19.57%)	20 (27.40%)	
Missing	46	12	24		21	15	
**Respiratory bacteria**[b]				0.905			0.843
no	174 (66.16%)	49 (68.06%)	80 (66.12%)		86 (67.72%)	43 (65.15%)	
yes	89 (33.84%)	23 (31.94%)	41 (33.88%)		41 (32.28%)	23 (34.85%)	
Missing	71	19	35		32	22	
**Helminths**[c]				0.970			0.724
Negative	222 (66.47%)	64 (70.33%)	108 (69.23%)		109 (68.55%)	63 (71.59%)	
Positive	112 (33.53%)	27 (29.67%)	48 (30.77%)		50 (31.45%)	25 (28.41%)	
**Season**				0.895			0.840
Short Rains (Oct. - Feb.)	120 (36.04%)	34 (37.36%)	61 (39.10%)		59 (37.11%)	36 (40.91%)	
Long Rains (March - May)	74 (22.22%)	24 (26.37%)	37 (23.72%)		40 (25.16%)	21 (23.86%)	
Dry (June - Sept.)	139 (41.74%)	33 (36.26%)	58 (37.18%)		60 (37.74%)	31 (35.23%)	
Missing	1	0	0		0	0	
**MTBC lineage**				0.675			0.829
L1	39 (17.33%)	8 (18.18%)	23 (18.25%)		17 (17.89%)	14 (18.67%)	
L2	8 (3.56%)	2 (4.55%)	4 (3.17%)		4 (4.21%)	2 (2.67%)	
L3	102 (45.33%)	16 (36.36%)	58 (46.03%)		39 (41.05%)	35 (46.67%)	
L4	76 (33.78%)	18 (40.91%)	41 (32.54%)		35 (36.84%)	24 (32.00%)	
Missing	109	47	30		64	13	
**TB Diagnostic delay**[d]				0.747			0.379
Delay <= 3 weeks	102 (30.54%)	30 (32.97%)	47 (30.13%)		46 (28.93%)	31 (35.23%)	
Delay >3 weeks	232 (69.46%)	61 (67.03%)	109 (69.87%)		113 (71.07%)	57 (64.77%)	
**Non-TB medication**				0.509			0.445
No	20 (5.99%)	7 (7.69%)	7 (4.49%)		11 (6.92%)	3 (3.41%)	
Penicillins	264 (79.04%)	72 (79.12%)	124 (79.49%)		126 (79.25%)	70 (79.55%)	
Other	50 (14.97%)	12 (13.19%)	25 (16.03%)		22 (13.84%)	15 (17.05%)	

[a] Associations of categorical demographic and clinical characteristics with TB disease manifestations were assessed by chi-squared test or Fisher's exact test(‡) if expected frequencies were below five. Associations with continuous variables were assessed by student t-tests or Wilcoxon rank-sum test (†) when normality could not be assumed. In bold, values below Bonferroni-adjusted significance criteria ($\alpha = 0.05/20$); [b] Anyplex^{TM} II RV16 and Allplex^{TM} Respiratory Panel 4, Seegene. 16 respiratory viruses and 6 bacterial species; [c] 8 helminth parasites: *Ascaris lumbricoides*, *Enterobius vermicularis*, hookworm, *Hymenolepis diminuta*, *Schistosoma haematobium*, *Schistosoma mansoni*, *Strongyloides stercoralis* and *Trichuris trichiura*; [d] Duration of diagnostic delay was calculated based on the longest reported TB-related symptom and categorized into: '≤3 weeks' and '>3 weeks'; [e] To screen for lung abnormalities, double readings of chest x-rays were performed by board-certified radiologists, and discrepancies were resolved by an independent reader; IQR, interquartile range; ART, antiretroviral therapy

REFERENCES
